# A systematic review and qualitative research synthesis of the lived experiences and coping of transgender and gender diverse youth 18 years or younger

**DOI:** 10.1080/26895269.2023.2295379

**Published:** 2024-01-12

**Authors:** Kristiina Tyni, Matilda Wurm, Thomas Nordström, Anna Sofia Bratt

**Affiliations:** a Department of Psychology, Linnaeus University, Växjö, Sweden; b School of Behavioral, Social and Legal Sciences, Örebro University, Örebro, Sweden

**Keywords:** Children and youth, gender identity, gender diverse, qualitative, systematic review, transgender

## Abstract

**Background:**

Research on the daily experiences of transgender and gender diverse (TGD) youth 18 years or younger is limited, making it essential to gain a comprehensive understanding of their internal and external experiences related to gender identity.

**Aim:**

This systematic review and qualitative research synthesis fills this research gap by examining the lived experiences and coping of TGD youth, including prepubertal children.

**Methods:**

The review was pre-registered according to PROSPERO on the Open Science Framework and followed the ENTREQ reporting guidelines. A Qualitative research synthesis, according to Howell Major and Savin-Baden, was conducted.

**Results:**

Seventeen peer-reviewed articles published between 2000 and 2023 fulfilled inclusion criteria and quality assessment. Synthesized themes were: (1) “Navigating gender identity”, with two sub-themes, *Meaning-making* and *Considering visibility* (2) “Navigating relations”, with four sub-themes: *Longing for belonging, Supportive actions, Lack of safety* and *Coping inside out* (3) “Navigating society with two sub-themes *Inclusion and exclusion* and *Beyond control*. Our findings demonstrate that TGD youth view gender identity as fluid and benefit from a supportive environment that facilitates genuine exploration. Coping strategies develop intricately, influenced by multifaceted factors.

**Discussion:**

Unlike previous research on the negative effects of minority stress, our review underscores the cumulative impact of subtle daily stressors on TGD youth’s well-being, highlighting the significance of an environment where gender is not a constant concern. By shedding light on these dynamics, this synthesis contributes to a comprehensive understanding of TGD youth’s perspectives for professionals and a broader audience.

## Introduction

Research on gender development and experiences of transgender and gender diverse (TGD) youth 18 years or younger is limited (Ehrensaft et al., 2018; Olson-Kennedy et al., 2016). Increasing numbers of TGD youth live as their identified genders from a young age, having made a social transition (i.e., change of name, pronoun, and gender expression) with the support of their parents (Ehrensaft et al., 2018). TGD youth encompasses young individuals who experience a discrepancy between their sex assigned at birth and their internal psychological sense of their gender and how they are perceived by others (American Psychological Association [APA], 2023; American Psychological Association [APA], 2015; Bränström, 2019). While some TGD individuals identify with a binary gender, others identify as nonbinary or genderqueer or have a fluid gender identity.

TGD youth, especially those identifying as nonbinary, report higher levels of depression symptoms, anxiety, self-destructive behavior, and suicidal ideation/attempts than their cisgender peers (Bränström, 2019; de Graaf et al., 2021). This is largely attributed to societal discrimination and stigma against gender diversity (Bränström, 2019; Johnson et al., 2023).

Gender dysphoria, the psychological distress caused by the discrepancy between one’s gender identity and sex assigned at birth (Coleman et al., 2022; Guss et al., 2015), can result also from societal pressure to conform to traditional gender norms (Bränström, 2019: McGowan et al., 2022; Monro, 2000). However, it is important to note that not all TGD individuals experience gender dysphoria (APA, 2023; APA, 2015).

In previous research, TGD youth supported and affirmed by their parents from an early age exhibited similar well-being as their cisgender counterparts in control groups (cisgender siblings and unrelated cisgender youth). Anxiety levels were somewhat heightened but far below clinical measures, possibly explained by the higher risk of stigma-related exposure that TGD youth face in their families, at school, and in society compared to cisgender youth (Durwood et al., 2017). This underscores the importance of parental support and affirming environments for TGD youth’s mental health and well-being (Grossman et al., 2021; Kuvalanka et al., 2017; Singh et al., 2014; Singh, 2013; Simons et al., 2013).

Transgender children with a binary gender (boy or girl) strongly identifiy as members of their identified gender group, and in research gender-typed preferences did not differ from unrelated, gender-matched cisgender children (Gülgöz et al., 2019). Both cisgender and transgender children’s gender developmental patterns also showed coherence across measures, and there were no or minimal differences regardless of the time children had lived as their current gender (Gülgöz et al., 2019). This suggests that gender identity, or how a child expresses their gender later, is not always based on the sex assigned to a child at birth (e.g., girl/boy) or parental rearing based on that sex (Gülgöz et al., 2019). Even if most children know their sex/gender from about 3 years of age (Perry et al., 2019), the developmental paths to an affirmed gender identity described by TGD youth are various (Medico et al., 2020; Pullen Sansfaçon et al., 2020; Steensma et al., 2011). When examining children’s beliefs about the inborn and stable nature of gender/sex, transgender children were less likely than unrelated cisgender children to assume that a child labeled “boy” was also born with a boy’s body parts and feeling like a boy, indicating that transgender children interpret gender as more ambiguous than their cisgender counterparts (Gülgöz et al., 2021).

Still, further research is needed to gain a comprehensive understanding of the experiences of TGD youth 18 years or younger, considering their dependence on caregivers and school environments as minors, distinct from adult transgender people (Ehrensaft et al., 2018; Gülgöz, 2019; Olson-Kennedy et al., 2016). Consequently, our review seeks to bridge this knowledge gap and provide insight into the internal and external experiences of TGD minors by focusing on their *lived experience* (i.e., personal, first-hand experiences in everyday life).

### Minority stress, microaggressions, and emotional labor

The *Minority stress model* (Meyer, 2003) offers a framework for understanding the negative impact of prejudice, discriminatory, or violent treatment on the physical and psychological well-being of people belonging to minority groups, including TGD individuals (Bränström & Pachankis, 2021; Hendricks & Testa, 2012; Meyer, 2015; Testa et al., 2017). However, criticism has been made that the minority stress theory does not sufficiently include all forms of exposure that transgender individuals may experience daily. For example, Nadal et al. (2012) point out that microaggressions, such as receiving strange looks or facing inappropriate personal questions due to prejudice or a lack of knowledge, can also be sources of long-term stress. In a Swedish qualitative study by Lundberg et al. (2023) focusing on the experiences and coping of 29 transgender adults, the concept of *emotional labor* (Hochschild, 1979) was useful in comprehending the emotional drainage of energy it meant to either conceal their gender identity or face daily instances of microaggressions. The study also shed light on how a supportive environment, offering a sense of relief, could lead to improved health and well-being (Lundberg et al., 2023). The concept of emotional labor refers to managing and regulating ones emotions to align with social expectations, even if they do not reflect onés true feelings. This kind of emotional effort can create emotional dissonance, where there is a disconnect between genuine and expressed emotions, leading to a potential decrease in self-esteem (Jeung et al., 2018). In an already stressful environment, emotional labor can turn the loss of resources in a strained situation into an even more stressful one (Jeung et al., 2018). Emotional labor has been extensively studied in workplace settings showing increases in stress levels, depressive symptoms, and the risk of burnout over time (Jeung et al., 2018; Maslach, 1982; Shankar & Kumar, 2014). Coping strategies and the availability of social support seem to moderate the relationship (Shankar & Kumar, 2014).

### Coping

The impact and handling of stressful events depend on how the situations are perceived. As TGD youth face a heightened risk of developing mental health problems due to minority stress compared to cisgender youth, gaining a deeper understanding of the intricate nature of coping as they navigate their gender identity journeys is crucial. The term *coping* from Lazarus and Folkman’s *Transactional model of stress and coping* is well-established in research referring to several stress management strategies (Lazarus & Folkman, 1984). Coping is often divided into *task-focused coping*, which directly handles a stressor and requires learning new skills and approaches, and *emotion-focused coping*, which regulates the emotions rising due to stressful situations, for example, by avoidance and distancing but also by seeking support, positive reevaluation, and acceptance. Task-focused coping is primarily employed when the source of stress is perceived as controllable, whereas emotion-focused coping is utilized in response to persistent stressors (Lazarus & Folkman, 1984). Flexibility in using different coping strategies depending on the context seems most helpful for stress management (Shankar & Kumar, 2014). In the 21st century, coping strategies have expanded to include new categories, such as *proactive coping*, aimed at foreseeing and preventing possible future stressors (Biggs et al., 2017). According to Biggs et al. (2017), the use of proactive coping has been shown to lessen burnout symptoms as it serves to collect experience and knowledge and, in a sense, actively plan the coping of possible future stressors in upcoming situations. A coping strategy, regardless of its nature, can either prove beneficial or detrimental depending on factors such as context, timing, and intention (Budge et al., 2018). Youth also observe, react, and learn from the different coping responses employed by their family members regarding situations and emotions linked to the youth’s gender identity (Budge et al., 2018; Katz-Wise et al., 2020).

As TGD youth navigate the crossroads of their gender identities and daily lives, the shadows of minority stress and emotional labor loom. Further research is needed to gain a comprehensive understanding of the internal and external experiences of TGD youth 18 years or younger related to gender identity (Ehrensaft et al., 2018; Gülgöz, 2019; Olson-Kennedy et al., 2016). Qualitative research, which focuses on the voices and viewpoints of participants and aims to provide a deeper understanding of their experiences, is particularly relevant in this context (Swedish Agency for Health Technology Assessment and Assessment of Social Services [SBU], 2020). Qualitative methods, such as interviews, enable researchers to explore complex issues and capture the diversity and nuances of people’s experiences. This systematic review aimed to synthesize existing qualitative research and provide a comprehensive understanding of the lived experiences of TGD youth 18 years or younger to inform and enhance knowledge in people meeting and working with TGD minors.

### Previous reviews

Previous reviews on TGD individuals have explored various topics, such as protective factors for health and well-being (Johns et al., 2018); transgender community connection and well-being (Sherman et al., 2020); accessing gender-affirming healthcare (Kearns et al., 2021); subjective experiences of gender dysphoria (Jessen et al., 2021); risk and resilience factors for mental health (Tankersley et al., 2021); and minority stress, coping, and resilience (Smith et al., 2021), as well as the lived experiences of transgender individuals (Moolchaem et al., 2015). However, most of these existing reviews have primarily focused on quantitative or mixed-method approaches, encompassing broader age ranges that differ from the scope of the current review. For instance, Tankersley et al. (2021) examined 44 quantitative studies involving transgender youth aged 3–24. Other reviews, including both quantitative and qualitative studies, encompassed participants aged 11–26 (Johns et al., 2018); 8–25 (Kearns et al., 2021); 7–61 years (Valentine & Shipherd, 2018). In Sherman et al. (2020) only two articles included participants as young as 15 years, while the rest featured individuals above 18 years.

In the reviews, specifically focusing on qualitative studies, participants’ age ranges were 12–29 years (Jessen et al., 2021), 14–74 years (Moolchaem et al., 2015), and 17–83 years (Smith et al., 2021). While Moolchaem et al. (2015) explored the same phenomenon as our review (i.e., lived experience), they only included two articles with a few minors in each. The adult transgender people in their review dealt with many experiences TGD youth in our included studies had not experienced as minors, for example, surgical transitions, sexually transmitted diseases such as HIV, and work-life experiences. Hence, the lived experiences of TGD youth 18 years or younger have not specifically been highlighted in previous reviews.

Of particular relevance is the study by Jessen et al. (2021), which identified four themes describing the experiences of individuals with gender dysphoria. The findings indicated that participants often perceived themselves as different from a young age as feeling a lack of congruence between their internal sense of gender and the external world, frequently resulting in distress (Jessen et al., 2021). The body played a crucial role in the subjective experience of gender dysphoria, but youth also experienced a sense of not feeling whole as they were being misrecognized by others. However, Jessen et al. (2021) did not differentiate the experiences of minors from those of older participants, making it challenging to exclusively ascertain knowledge about the experiences of children and youth.

To date, no prior reviews have specifically explored the experiences and coping mechanisms related to gender identity in TGD youth 18 years or younger. Therefore, our objective was to synthesize qualitative research within this field, aiming to explore TGD youths’ lived experiences pertaining to gender identity and their coping strategies. In the review, both external experiences, such as relational events, and internal experiences, like figuring out gender identity, were included.

### Research questions

1. What are the lived experiences of TGD youth 18 years and younger related to gender identity?
2. How do they cope with their experiences?

## Methods

### Synthesis methodology and approach to literature search

This review examines subjective narratives of lived experiences. Therefore, all the studies included in the review are qualitative and provide first-hand interview data. The synthesis of the selected qualitative studies was conducted according to the guidelines formulated by Howell Major and Savin-Baden (2010) for doing a Qualitative Research Synthesis.

We adhere to a philosophical framework of critical realism (Bhaskar, 2008), often used in social research, including transgender research (Pilgrim, 2022). It seeks to understand the underlying structures and mechanisms that shape social phenomena, acknowledging that reality is not directly accessible but shaped by observable events and underlying causes. In the context of transgender research, critical realism emphasizes the need to explore both the observable experiences of transgender individuals and the deeper social, cultural, and psychological factors that influence their gender identities and experiences. This approach aims to uncover the complex interplay between individual agency and broader social structures, providing a more comprehensive understanding of transgender issues. Regarding TGD youth, the authors of this study adopt the position that being transgender is a phenomenon that exists, acknowledging and affirming the identities of TGD children and teens. We believe that various contextual factors, such as family dynamics, school environments, and societal norms, shape the expression and individual experiences of TGD youth. Three of the manuscript’s authors are experienced in conducting systematic reviews, and two have conducted qualitative syntheses. Within the research group, there are individuals with diverse experiences in terms of gender identity and sexual orientation.

For reporting, we followed The Enhancing Transparency in Reporting the Synthesis of Qualitative Research (ENTREQ) statement (Tong et al., 2012). The study was preregistered in January 2021 at the Open Science Framework (OSF) for research transparency: http://dx.doi.org/10.17605/OSF.IO/9TUH3.

### Eligibility criteria

Inclusion criteria: (1) population: TGD youth 18 years or younger; (2) English language; (3) studies published between the years 2000–2023; (4) peer-reviewed journals; (5) qualitative studies including first-hand interview data of experiences and coping related to gender identity. If mixed methods were used, we only included the qualitative part of the results since we focus on youth’s lived experiences.

Studies were excluded if any of the inclusion criteria were not fulfilled but also if interview data were not reported and analyzed separately from (1) non-TGD participants, for example, sexual minorities; (2) TGD individuals older than 18 years; (3) secondary data about TGD youth (i.e., from parents, caregivers, or others)

### Search strategy and study selection

A comprehensive, pre-planned, standardized search method was used to seek all available studies in the field. The first author (KT) and a specialized librarian at Linnaeus University conducted the literature searches. Electronic databases in the intersection of psychology, sociology, and medicine/psychiatry—PsycINFO, ASSIA (ProQuest), and PubMed—were searched to capture articles about TGD identities 18 years or younger; experiences and coping; qualitative research; and qualitative analysis methods. The literature searches were done separately, one database at a time, to use both keywords and controlled thesaurus search terms since the latter vary in the different databases. See Supplementary Appendix A for information on search strings. An updated search was done on the 30th of March, 2023. Grey literature was searched by generic web searches using Google Scholar, of which the first 200 results were examined (Bramer, 2016). In addition, the site ResearchGate was used for communication with researchers and for keeping updated on new publications during the study.

### Study screening methods

**Figure 1.**
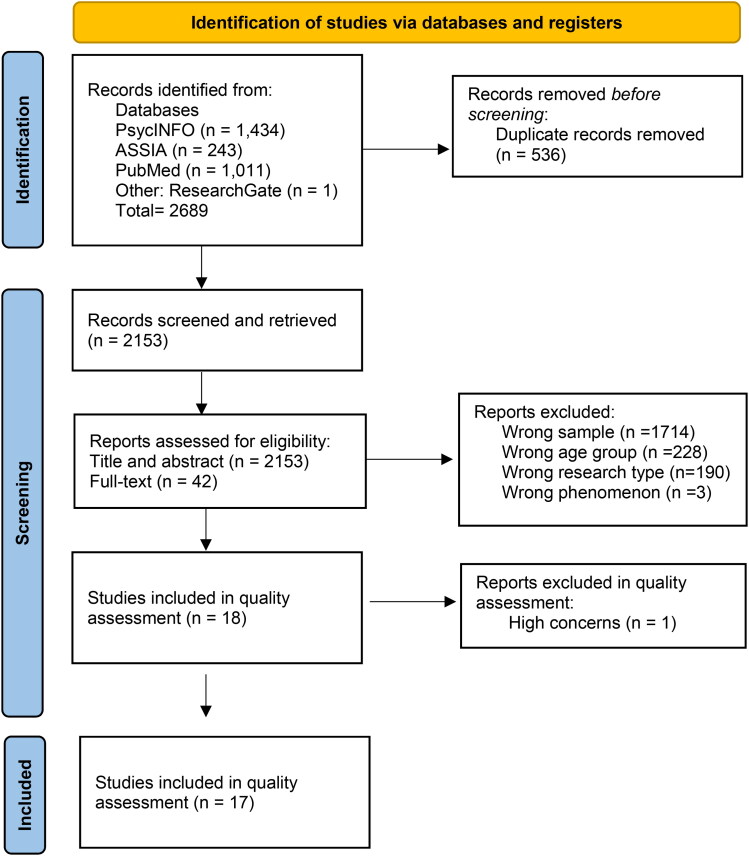
PRISMA 2020 flow diagram.

The search tool Sample, Phenomenon of Interest, Design, Evaluation, and Research type (SPIDER), developed for qualitative studies, was used for the screening (Cooke et al., 2012; Methley et al., 2014). All four authors participated in the screening process after the first search. The Zotero software 6.0.26 (Courraud, 2014), and Rayyan (Ouzzani et al., 2016) for the updated selection, were used as a shared tool between the authors for organizing and screening for duplicates during the selection phase. After duplicates were removed, 2153 records were divided between all four authors and screened to check for eligibility criteria according to our SPIDER. The title and abstract were read independently by the reviewers, and 42 articles were read in full text, of which 24, with possible question marks, were checked between two authors (KT and ASB) regarding the type of research and design, but there were no disparities. See Figure 1. PRISMA flow diagram in the Results section for more information on screening the process.

### Study selection

Authors KT and ASB considered the quality of the studies according to the guidelines from the SBU (2020). These national guidelines are based on the Cochrane tool for assessment of the quality of qualitative research, GRADE-CERQual. The SBU guidelines include five domains for quality assessment: (1) theoretical and philosophical framework related to research question/aim; (2) population selection—sample and recruitment; (3) data collection; (4) data analysis; and (5) the role/positionality of the researcher. Each domain includes questions as guidance in the assessment—see Supplementary Appendix B for an English translation of all questions. The two authors (KT and ASB) separately checked each article and made a final overall assessment of possible risks for the identified shortcomings possibly skewing the study’s results, ranging from (1) insignificant concerns, (2) moderate concerns, and (3) high concerns, according to the guidelines (SBU, 2020). One article was excluded due to high concerns after the final overall assessment; see Table 1 in the Results section for relevant assessment information of included/excluded studies related to the SBU domains.

**Table 1.** Quality assessment information according to the SBU guidelines.

Authors (year)	Phenomenon of interest	Theoretical /philosophical stance, analysis method	Age-span	Sample	Recruitment	Researchers’ positionality	Ethical approval	Assessment summary, including missing aspects and a final evaluation of inclusion/exclusion
Budge et al. (2018)	Coping related to gender identity by conceptualizing coping as a developmental process—what thoughts and actions do trans youth describe as helpful throughout the various developmental aspects of their gender identity processes?	Grounded theory analysis (Charmaz, 2014). Social constructionism.	7–18	*N* = 20 transgender and gender diverse (TGD) youth. Youth self-identified their current gender identity as a trans boy (12), trans girl (6), gender fluid boy (1), and girlish boy (1). White (90%), multiracial (10%).	*via* support networks and LGBTQ community organizations for families with transgender youth, and snowball sampling. The study is part of a larger qualitative study—the Trans Youth Family Study.	Yes	Yes	**Included (insignificant concerns).** The interview guide was not included, but examples of questions asked were specified.
Budge et al. (2021):	Emotions related to gender identity; how TGD youth understand, experience, and label their emotional experiences	Same as above, except for the second phase of the analysis included deductive coding.	7–18	The same as above, Budge et al. (2018).	The same sample as Budge et al. (2018)	Yes	Yes	**Included (insignificant concerns).** The interview guide was not included, but examples of questions asked were specified.
Clark et al. (2020):	Trans youth’s (and parents’) decision-making around hormone therapy initiation and experiences of barriers to care	Grounded theory analysis (Charmaz, 2014). Social constructivism.	14–18	*N* = 21 trans youth identifying within the three categories of trans youth female or transfeminine (8); male or transmasculine (8), and non-binary or genderfluid (5), and 15 parents to trans youths. Race/ethnicity was not specified.	Recruited through purposive sampling, with additional snowball sampling to elicit diverse perspectives from organizations serving trans youth and their parents (e.g., health clinics, community organizations, support groups. The study is part of a larger research project on trans youth hormone therapy decision-making.	No	Yes	**Included (moderate concerns).** No interview guide was mentioned or included. Nothing on security or anonymization. No positionality of researchers’.
Clark & Virani (2021):	Transgender youth capacity, rights, and authority to consent to hormone therapy	Content analysis (Hsieh and Shannon 2005). Social constructivism and critical realism.	14–19	*N* = 21 trans youth, no further info	Recruited through healthcare organizations, community organizations, and community events that served trans youth, parents of trans youth, and healthcare providers working with trans youth (in this review, only the results from youth were included). Participants were part of a larger qualitative research project, the Trans Youth Hormone Therapy Decision-Making Study	No	Yes	**Excluded (high concerns).** Only the age of participants was specified (not gender identities, ethnicity, or other information). No interview guide or examples of questions were included, nor who did the interviews and analysis. Reflexiveness of the analysis process was missing. Nothing on security or anonymization. No positionality of researchers’.
Durwood et al. (2022)	Retransition experience	Thematic analysis (Braun & Clark, 2006). No theoretical/philosophical stance.	(9–13?) *M*:11,3	*N* = 10 initially, binary transgender youth who retranstitioned (used some other pronoun than their initial transgender one) at least once before 1 Jan 2021. When interviewed: cisgender (3), nonbinary (5), binary transgender (2; after having transitioned to a nonbinary identity). The majority were white	The participants were part of the Trans Youth Project, a longitudinal study of 317 initially binary, socially transitioned (use some other pronoun than the one given at birth at/before the age of 12) transgender children recruited between 2013 and 2017 through various channels, for example, gender clinics, camps for gender-diverse children, support groups and media stories.	No	Yes	**Included (insignificant concerns).** The age of participants at the time of interviews was not specified more than the ones in the citations. Still, the ages of retransitions were specified in a separate table. The interview guide was not included, but the structure, procedure, and who conducted the interviews were specified. No epistemological stance nor researchers’ positionality.
Katz-Wise et al. (2020)	Family- and community-based experiences related to TNBC youth identity.	Thematic analysis (Clark & Braun, 2006) and a family and ecological systems approach.	13–17	*N* = 33 transgender and non-binary (TNB) youth identified as trans girls (12), trans boys (17), and nonbinary (3 assigned female at birth and 1 assigned male at birth). White (73%), mixed race/ethnicity (15%).	From community-based venues, including support organizations, youth drop-in centers, LGBTQ organizations, homeless shelters, medical and mental health providers, and gender clinics. This study is part of a larger longitudinal, mixed-method study (the Trans Teen and Family Narratives Project, TTFN).	. No	Yes	**Included (insignificant concerns).** The interview guide was not included, but examples of questions asked were specified. Nothing on security or anonymization. No positionality of researchers.
Kelley et al. (2022)	Lived experiences and coping of TNB youths in a school setting and its impact on well-being and resilience	Thematic analysis (Clark & Braun, 2006) and a minority stress theoretical framework.	15–17	*N* = 12 participants identified as trans girls (2), trans boys (4), and nonbinary (6). White (11), mixed heritage (1).	Participants were recruited *via* community partner organizations using paper (posters and flyers) and online invitations. All were attending high school in Quebec. In-person, one-on-one interviews, 2 h, in location and language (Eng/French) chosen by participants. This data was drawn from a bigger Canadian project (*N* = 54), 15–25-year-old TNB youth.	No	Yes	**Included (insignificant concerns).** No interview guide or examples of questions are included. No philosophical stance or reflexivity on the researchers’ positionality.
Leonard (2022)	Positive school experiences of transgender children and young people (CYP)	Interpretative phenomenological analysis (Smith et al., 2009). Reflexive on philosophical stance.	16–18	*N* = 3 transgender youth; identified as male (2) and transwoman (1). Race/ethnicity was not specified.	Participants were recruited from an LGBT youth group *via* purposive and snowball sampling. One-on-one, one-time interviews, 40–70 min.	Yes	Yes	**Included (insignificant concerns).** A small sample, but the analysis method chosen is appropriate for a small number of participants. No interview guide or examples of questions are included.
McGowan et al. (2022)	Experiences of transgender young people in secondary education	Inductive thematic analysis (Braun & Clark, 2006). Social constructionism.	Unclear: in Abstract 11–16 but in Method 13–16	*N* = 10 transgender young people; male (8), female (1), either male or female (1).	Recruitment through opportunity sampling. All participants attended a charity weekend led by Mermaids, a charity supporting young transgender or gender variant people and their families.	Yes	Yes	**Included (insignificant concerns).** All participants were recruited from the same charity weekend (a charity supporting trans people and families). They still lived in different places, attended different secondary schools.
McKenna et al. (2022)	Transgender and gender diverse adolescents’ use of video game avatar creation for gender-affirmation and exploration.	Thematic analysis (Braun & Clark, 2006). No theoretical/philosophical stance.	13–18	*N* = 10 transgender and gender diverse (TGD) adolescents self-identifying as: male (3), transmasculine (1), girl (1), transgender female (3), nonbinary (2), nonbinary demiboy (1), and had experience creating avatars while playing video games. White (8), Asian (1), African American (1).	Participants were recruited through a gender clinic in Massachusetts.	Yes	Not mentioned any.	**Included (moderate concerns).** No interview guide was included, but areas covered in the interview were described. Ethical approval not specified but since recruitment was approved by the gender clinic we assume there was an approval? No theoretical/philosophical stance.
Pullen Sansfaçon et al. (2019)	The experiences of gender diverse and trans children and youth considering and initiating medical interventions	Inductive thematic analysis (Braun & Clark, 2006), guided by the perspective of social determinants of health. No philosophical stance.	9–17	*N* = 35 trans youth; 14 youth were transfeminine (TF), and 22 were transmasculine (TM). All youth described their identity in binary terms, except for three transmasculine youth (1 non-binary, 2 genderfluid). White (31), nonwhite or Indigenous (4).	The youth sample was drawn from a larger research project where recruitment and data collection took place in three Canadian clinics offering gender-affirming care. Both youth and parent/-s had to participate, but in the article, it is the youth sample only.	No (except for the gender identity of the research assistant conducting interviews and analysis under the supervision of the lead researcher).	Yes	**Included (insignificant concerns).** No interview guide was included, but areas covered in the interviews were described. No epistemological stance nor researchers’ positionality.
Pullen Sansfaçon et al. (2020)	Trans youth’s experience of considering and initiating medical interventions.	Grounded theory (Strauss & Corbin, 1998) and Thematic analysis (Braun & Clark, 2006), using MAXQDA data analysis software. No theoretical/epistemological stance.	9–17	*N* = 36 trans youth. The same sample as Pullen Sansfaçon et al. (2019), but 1 more (36) participant.	Same as above—Pullen Sansfaçon et al. (2019).	No	Yes	**Included (moderate concerns).** No interview guide or examples of questions. Who did the analysis was not specified. No theoretical/epistemological stance nor researchers’ positionality.
Saltis et al. (2023)	How transgender and gender expansive youth (TGEY) experience and cope with oppression	Interpretative phenomenological analysis with subjectivist epistemology situated within interpretivism, critical theory, and intersectionality theory.	13–17	*N* = 9 transgender and/or gender expansive youth (TGEY); Male (2), Female (2), Trans-female (1), Gender-fluid (3), Agender (1). Caucasian and Native American, Caucasian, White, Caucasian, White, White European, Caucasian and Chicano, Caucasian/Finnish, Bi-Racial, Mexican Caucasian	Purposeful sampling and snowball sampling through distributing a flyer to organizations, social media pages, and community agencies allied with TGEY.	Yes	Yes	**Included (insignificant concerns).** No interview guide and no examples of questions.
Selkie et al. (2020)	Transgender adolescents’ uses of social media for social support.	Thematic analysis (no refence) with initial coding *via* NVivo software. No theoretical/philosophical stance.	15–18	*N* = 25 transgender adolescents; transmasculine (13), transfeminine (11), and nonbinary (1). White non-Hispanic (20), African American (1), American Indian (2), Asian (2).	Transgender adolescents with a social media profile were recruited from a pediatric gender clinic.	No	Yes	**Included (insignificant concerns).** Mentions general thoughts about reflexivity/positionality of researchers but still no specifics. No theoretical/philosophical stance declared.
Steensma et al. (2011)	The desisting and persisting of gender dysphoria after childhood.	“the method of open coding” (no reference) using the ATLAS.ti v5.2 software for initial coding. No theoretical/philosophical stance.	14–18	*N* = 25 adolescents diagnosed with a Gender Identity Disorder (DSM-IV or DSM-IV-TR) in childhood; 14 adolescents (7 boys and 7 girls) who applied for sex reassignment at the Gender Identity Clinic in adolescence (*M* age 16.0, range 14–18) and 11 adolescents (6 boys and 5 girls) who had no further contact with the clinic after childhood (*M* age 15.72, range 14–18). Not clear if the sex mentioned was the sex assigned at birth or gender identity, nor if social transition was ever fulfilled. Race/ethnicity was not specified.	Participants were part of a larger study at the Gender Identity Clinic at the Amsterdam VU University Medical Center (VUmc). Of 198 children referred to the Gender Identity Clinic at the Amsterdam VU University Medical Center (VUmc) when <12 years old, 53 adolescents were offered to participate in this study when they were >14 years old.	No	Yes	**Included (moderate concerns).** Gender identity (?) and race/ethnicity not specified. No reference or name of the analysis method was specified. No interview guide and no examples of questions. Nothing on security or anonymization. The analysis process was described but no name of a regular method. No philosophical stance nor researchers’ positionality.
Vrouenraets et al. (2016)	The considerations of adolescents with gender dysphoria in the Netherlands concerning the use of puberty suppression.	“the constant comparative method.” (no name of method or reference). The analysis process was described, but no specific analysis method. No theoretical/philosophical stance.	13–18	*N* = 13 adolescents diagnosed with Gender dysphoria.; 5 trans girls (5) and trans boys (8). No race/ethnicity was specified.	The informants were recruited from the Gender Identity clinic in Leiden. The interviews were conducted in the context of a larger study where both adolescents and professionals working in treatment teams were interviewed and data compared. In this article, it’s the children’s data only.	No	Yes	**Included (moderate concerns).** Race/ethnicity was not specified. No interview guide or example of questions included. The title and education of the interviewer were mentioned but not for researchers conducting the analysis, with no reflexivity of pre-understanding. The analysis process was described, but not any specific analysis method nor theory or epistemological position was specified. Nothing on security or anonymization.
Weinhardt et al. (2019)	Experiences of social support and how it relates to well-being and resilience among transgender youth.	Thematic analysis (Saldaña, 2016), with Deedose (2016) software for initial coding. Approach and rationale described but no theoretical/philosophical stance.	14–17	*N* = 8 transgender and gender diverse youth (TGD) transgender (37.5%), nonbinary/gender fluid (25%), or multiple gender identities (37.5%). 7 individuals were assigned female at birth, and 1 individual was assigned male at birth. Non-Hispanic White (65.6%), Hispanic or Latina (3.2%), African American (4.5%, Asian American (2.6%), and multiracial (24%).	Interview data were collected from a focus group at a week-long leadership development camp for high-school-age LGBT youth. The LGBT resource center on the campus of a local university organized the camp.	No	Yes	**Included (moderate concerns).** No interview guide or example of questions included. The approach and rationale of analysis were described but no philosophical stance nor researchers’ positionality.
Wilson et al. (2005)	The participants’ interaction with their peers in secondary school.	Thematic analysis (Smith, 1995). No theoretical/philosophical stance.	14–17	*N* = 8 children with Atypical gender identity organization; male to female (6) (MtF), female to male (2) (FtM]). Race/ethnicity was not specified.	Participants were recruited at a Gender Identity Development Unit (GIDU).	No	Yes	**Included (moderate concerns).** Race/ethnicity was not specified. No interview guide was included, but two main questions were specified. Nothing on security or anonymization. Who conducted the interviews and analysis was mentioned, but nothing on researchers’ positionality or philosophical stance.

Final evaluation: Insignificant (inclusion); moderate (inclusion); high concerns (exclusion).

### Data analysis

The data analysis followed the procedure for Qualitative Research Synthesis described by Howell Major and Savin-Baden (2010). The authors KT and ASB conducted the analysis and the final synthesis, working independently but meeting regularly for reflexive discussions. Initially, a summary was made to compare the selected studies by their characteristics and main themes (see Table 2). Study Features in Results). The results sections in the included studies were used as our data for synthesis, and these sections were carefully examined to identify and extract categories and concepts aligning with our research questions. The extracted data were then identified for common themes across studies, so-called first-order themes, using an inductive approach. Related first-order themes were analyzed and reduced to a minor number of second-order themes in an iterative, dynamic process of arranging and rearranging for clear second-order themes to appear. Connections, similarities, and differences among the studies were considered, and the different contexts, nuances, and contradictions were explored. Finally, we synthesized and developed third-order interpretations by revisiting and rethinking important patterns and connections in the first and second-order themes. The themes and sub-themes of this synthesis, describing a new whole, were finally summarized as text with relevant quotes showing the themes were grounded in data. The third-order interpretations were validated by authors MW and TN to ensure the credibility and trustworthiness of the synthesis.

**Table 2.** Features of the 17 primary studies included in the qualitative research synthesis.

No.	Authors (year)	Title	Country	Context of data collection	Sample (sample size, category, gender identity, race/ethnicity)	Age	Phenomenon of interest	Results/themes	Research design
1.	Budge et al. (2018)	A grounded theory study of the development of trans youths’ awareness of coping with gender identity	United States of America (USA; Southern, Midwestern, and Northeastern—New England—areas).	Recruitment occurred *via* support networks and LGBTQ community organizations for families with transgender youth and snowball sampling. Individual interviews at the participants’ homes or in private offices at the researchers’ institutions. The study is part of a larger qualitative study—the Trans Youth Family Study—aimed at understanding how TGD youth and their caregivers make sense of their emotional experiences, coping processes, relationships, identity processes, and future experiences.	*N* = 20 transgender and gender diverse (TGD) youth. Youth self-identified their current gender identity as a trans boy (12), trans girl (6), gender fluid boy (1), and girlish boy (1). White (90%), multiracial (10%).	7–18	Coping related to gender identity by conceptualizing coping as a developmental process—what thoughts and actions do trans youth describe as helpful throughout the various developmental aspects of their gender identity processes?	Negotiating gender Avoidance Emotional relief Personal solace Support Active engagement.	A constructivist grounded theory analysis (Charmaz, 2014).
2.	Budge et al. (2021)	Transgender and gender diverse youths’ emotions the appraisal, valence, arousal model	The same as above, Budge et al. (2018).	The same as Budge et al. (2018).	The same sample as Budge et al. (2018)	7–18	Emotions related to gender identity; how TGD youth understand, experience, and label their emotional experiences.	Results presented as an appraisal, valence, and arousal theory of emotions. Within the theory, emotions are categorized into four quadrants: Reflective/Unpleasant Anticipatory/Unpleasant Reflective/Pleasant Anticipatory/Pleasant	A constructivist grounded theory analysis (Charmaz, 2014). The second phase of the analysis included deductive coding.
3.	Clark et al. (2020)	Hormone therapy decision-making processes: Transgender youth and parents	Canada (British Colombia)	Recruitment through organizations serving trans youth and their parents (e.g., health clinics, community organizations, support groups). Individual interviews and lifeline drawings were held at locations selected by participants (e.g., home, health care clinic, library). The study is part of a larger research project on trans youth hormone therapy decision-making.	*N* = 21 trans youth identifying within the three categories of trans youth female or transfeminine (8); male or transmasculine (8); and non-binary or genderfluid (5) and 15 parents to trans youths. Race/ethnicity was not specified.	14–18	Trans youth and parents of trans youth decision-making around hormone therapy initiation as well as trans youth experiences of barriers to care.	A three-phase temporal model: Discovery (inter)action while seeking care Reflection after hormone therapy initiation.	A constructivist grounded theory analysis (Charmaz, 2014).
4.	Durwood et al. (2022)	Retransitioning: The experiences of youth who socially transition genders more than once	USA, Canada	In the sample of 15 families, 10 children participated in interviews. The participants were part of the Trans Youth Project, a longitudinal study of 317 initially binary, socially transitioned (use some other pronoun than the one given at birth at/before the age of 12) transgender children recruited between 2013–2017 through various channels, for example, gender clinics, camps for gender-diverse children, support groups and media stories. Interviews were held one-on-one by a research team member, mostly over the phone, but a few in the families’ homes.	*N* = 10 initially, binary transgender youth who retranstitioned (used some other pronoun than their initial transgender one) at least once before 1 Jan 2021. When interviewed: cisgender (3), nonbinary (5), binary transgender (2; after having transitioned to a nonbinary identity). The majority were white.	9–13	Retransition experience	Antecedents of social transitions: (1) Evolving gender identities (2) Social inputs to gender Others’ reactions to youth’s social transitions: (3) Acceptance (4) Relief (5) (Society’s) difficulty outside the binary General reflections on the multiple transitions journey: (6) Importance of support from, and narratives by, the trans (and trans-affiliated) community (7) Reflections on the initial transition decision (in the light of later transitions) (8) Follow the child’s lead	Thematic analysis (Braun & Clark, 2006).
5.	Katz-Wise et al. (2020)	Using family and ecological systems approaches to conceptualize family- and community-based experiences of transgender and/or nonbinary youth from the trans teen and family narratives project	USA (New England)	Participants were recruited from community-based venues, including support organizations, youth drop-in centers, LGBTQ organizations, homeless shelters, medical and mental health providers, and gender clinics. The individual interviews were held either in a private room at the researcher’s office, at the family’s home, or *via* teleconference. This study is part of a larger longitudinal, mixed-method study (the Trans Teen and Family Narratives Project, TTFN).	*N* = 33 transgender and non-binary (TNB) youth identified as trans girls (12), trans boys (17), and nonbinary (3 assigned female at birth and 1 assigned male at birth). White (73%), mixed race/ethnicity (15%).	13–17	Family- and community-based experiences related to youth TNB identity.	Eight themes corresponding to different levels of the Ecological systems model: individual level: identity processes emotions/coping family level general family experiences family support community level: general community experiences community support LGBTQ community societal/institutional level: external forces	Thematic analysis (Braun & Clark, 2006).
6.	Kelley et al. (2022)	School factors strongly impact transgender and non-binary youths’ well-being	Canada	Participants were recruited *via* community partner organizations using paper (posters and flyers) and online invitations. All were attending high school in Quebec. Participants chose location and language (Eng/French). This data was drawn from a bigger Canadian project (*N* = 54), 15–25-year-old TNB youth.	*N* = 12 participants; identified as trans girls (2), trans boys (4), and nonbinary (6). White (11), mixed heritage (1).	15–17	Lived experiences and coping of TNB youths in a school setting and the impact on well-being and resilience.	Obstacles and protective factors impacting TNB youth wellbeing Strategies used by TNB students to face adversity	Braun and Clark’s thematic analysis (2003), and in accordance with minority stress theory (Meyer, 2003).
7.	Leonard (2022)	“It was probably one of the best moments of being trans*, honestly!”: Exploring the positive school experiences of transgender children and young people	United Kingdom (UK)	Participants were recruited from an LGBT youth group *via* purposive sampling and, after that, through snowballing.	*N* = 3 transgender youth; identified as male (2) and transwoman (1). Race/ethnicity was not specified.	16–18	Positive school experiences of transgender children and young people (CYP)	The importance of language Individual teacher support Whole-school approaches The importance of community My own best friend	Interpretative Phenomenological Analysis, Smith et al. (2009).
8.	McGowan et al. (2022)	Living your truth: Views and experiences of transgender young people in secondary education	UK	Recruitment through opportunity sampling, all participants attended a charity weekend led by Mermaids, a charity supporting young transgender or gender variant people and their families.	*N* = 10 transgender youth; identified as male (8), female (1), either male or female (1). Race/ethnicity was not specified.	In abstract 11–16 but in methods section 13–16?	Experiences of transgender youth in secondary education	Seeking acceptance and validation Receiving acceptance and validation Active rejection and invalidation Passive rejection and invalidation Consequences of rejection and invalidation.	Inductive reflexive thematic analysis, Braun & Clarke (2006).
9.	McKenna et al. (2022)	“You can’t be deadnamed in a video game”: Transgender and gender diverse adolescents’ use of video game avatar creation for gender-affirmation and exploration	USA (northeast)	Participants were recruited through a gender clinic in Massachusetts, and interviews were held digitally (Zoom) by the first author.	*N* = 10 transgender and gender diverse (TGD) adolescents self-identifying as: male (3), transmasculine (1), girl (1), transgender female (3), nonbinary (2), nonbinary demiboy (1), and had experience creating avatars while playing video games. White (8), Asian (1), African American (1).	13–18	Experiences with avatar customization related to gender identity development.	Video game avatar customization offers a low stakes environment for gender exploration avatars offer both internal and external validation of gender aspirational appearance goals avatars allow players to enact aspirational appearance goals video games and avatars offer immersion and escapism for TGD adolescents.	Braun and Clark’s thematic analysis (2003).
10.	Pullen Sansfaçon et al. (2019)	The experiences of gender diverse and trans children and youth considering and initiating medical interventions in Canadian gender-affirming specialty clinics	Canada	Recruitment and data collection took place in three Canadian clinics offering gender-affirming care: Meraki Health Center (Montreal, Province of Quebec), the Children’s Hospital of Eastern Ontario (CHEO Ottawa, Province of Ontario) and the Health Sciences Center Winnipeg (Winnipeg, Province of Manitoba). The study is part of a larger research project with 35 dyads of trans youth and their parents (12 young people at each two gender health clinics and 11 at a third clinic). At the time of their interview, the youth had been receiving care at the clinic for a period ranging from one month to 6 years. In this article, it is the youth sample only.	*N* = 35 trans youth; 14 youth were transfeminine (TF), and 22 were transmasculine (TM). All youth described their identity in binary terms, except for three transmasculine youth (1 non-binary, 2 genderfluid) White (31), nonwhite or Indigenous (4)	9–17	Experience of considering and initiating medical interventions.	Four recurring themes were identified around youth access and experiences of medical intervention: path to access care desired medical interventions and expectations outcomes of the medical interventions overall experiences with clinical care and service received	Following grounded theory (Strauss & Corbin, 1998) for data collection, but thematic analysis (Braun & Clark, 2006) for data analysis.
11.	Pullen Sansfaçon et al. (2020)	“I knew that I wasn’t cis, I knew that, but I didn’t know exactly”: Gender identity development, expression and affirmation in youth who access gender-affirming medical care	Canada	The study is part of the same research project as above—Pullen Sansfaçon et al. (2020)—but 1 more dyad (36) of trans youth and parent-/s. In this article, it’s the youth sample only.	*N* = 36, the same sample as Pullen Sansfaçon et al. (2019), but 1 more (36) participant. All youth described their identity in binary terms, except for three transmasculine youth (1 non-binary, 2 genderfluid)	9–17	How gender identity and gender dysphoria are experienced, expressed, and addressed by youth who have started, or are just about to start, a gender-affirming medical intervention.	Two interlinked dimensions of the youth’s lives allow meaning-making of their gender identity: Internal or personal Interactional or social processes. Careful analysis reveals three gender identity development pathways that may be taken by youth, from early questioning to the affirmation of their gender identity: (1) Early dissonance, early affirmation, and transition (2) Early dissonance, delayed transition (3) Late appearance of gender dysphoria	Thematic analysis (Clarke & Braun, 2006).
12.	Saltis et al. (2023)	How transgender and gender expansive youth cope with oppression: Making sense of experiences and resiliency factors		Purposeful sampling and snowball sampling through the distribution of a flyer to organizations, social media pages, and community agencies allied with TGEY. Semi-structured interviews and one member checking meeting through Zoom.	*N* = 9 transgender and/or gender expansive youth (TGEY); Male (2), Female (2), Trans-female (1), Gender-fluid (3), Agender (1). Caucasian and Native American, Caucasian, White, Caucasian, White, White European, Caucasian and Chicano, Caucasian/Finnish, Bi-Racial, Mexican Caucasian.	13–17	How TGEY experience and cope with oppression	Resiliency factors: Creativity a/Music b/Arts and writing c/Creative hobbies d/ Performing/acting Activism and education a/ Specific acts of advocacy and/or activism the participants were involved in b/ Fighting against dehumanization c/The meaning made from this work d/ The shadow side of their work	Interpretative phenomenological analysis, Smith et al. (2009) situated within interpretivism, critical theory, and intersectionality theory.
13.	Selkie et al. (2020)	Transgender adolescents’ uses of social media for social support	USA (Midwestern area)	Transgender adolescents with a social media profile were recruited from a pediatric gender clinic in the Midwestern U.S.	*N* = 25 transgender adolescents; transmasculine (13), transfeminine (11), and nonbinary (1). White non-Hispanic (20), African American (1), American Indian (2), Asian (2).	15–18	Transgender adolescents’ uses of social media for social support	Emotional support (through peers and role models) Appraisal support (for validating their experiences) Informational support (for navigating health decisions and educating family and friends) Negative social media experiences (including harassment and exclusionary behavior online)	Thematic analysis (no reference).
14.	Steensma et al. (2011)	Desisting and persisting gender dysphoria after childhood: A qualitative follow-up study	The Netherlands (Amsterdam)	Of 198 children referred to the Gender Identity Clinic at the Amsterdam VU University Medical Center (VUmc) <12 years, 53 adolescents were selected to participate in this study when they were >14 years. The interviews were held either at the VUmc or if the adolescents did not want to travel to Amsterdam, at their homes.	*N* = 25 adolescents diagnosed with a Gender Identity Disorder (DSM-IV or DSM-IV-TR) in childhood; 14 adolescents (7 boys and 7 girls) who applied for sex reassignment at the Gender Identity Clinic in adolescence (*M* age 16.0, range 14–18) and 11 adolescents (6 boys and 5 girls) who had no further contact with the clinic after childhood (*M* age 15.72, range 14–18). Race/ethnicity was not specified.	14–18	The developmental trajectories of childhood gender dysphoria and the psychosexual outcome of children with gender dysphoria	Persistence of childhood gender dysphoria Desistence of gender childhood gender dysphoria	“the method of open coding” (no reference).
15.	Vrouenraets et al. (2016)	Perceptions of sex, gender, and puberty suppression: A qualitative analysis of transgender youth	The Netherlands (Leiden)	The informants were recruited from the Gender identity clinic in Leiden. The interviews were conducted in the context of a larger study on controversies surrounding puberty suppression in adolescents, where both adolescents and professionals working in treatment teams were interviewed and data compared. In this article, it’s the children’s data only.	*N* = 13 adolescents diagnosed with Gender dysphoria.; 5 trans girls (5) and trans boys (8). Race/ethnicity was not specified.	13–18	The considerations of adolescents with gender dysphoria in the Netherlands concerning the use of puberty suppression.	The difficulty of determining an appropriate lower age limit for starting puberty suppression. The lack of data on the long-term effects of puberty suppression. The role of the social context, for which there were two subthemes: (a) increased media attention, on television, and on the Internet (b) an imposed stereotype	The constant comparative method (Malterud, 2001; Strauss & Corbin, 1998).
16.	Weinhardt et al. (2019)	The role of family, friend, and significant other support in well-being among transgender and non-binary youth	USA (Midwestern area)	Interview data were collected from a focus group (8 participants) in a mixed-method study (survey and focus group). The focus group was held in a private room at a week-long leadership development camp for high-school-age LGBT youth. The LGBT resource center on the campus of a local university organized the camp.	*N* = 8 transgender and gender diverse youth (TGD) transgender (37.5%), nonbinary/gender fluid (25%), or multiple gender identities (37.5%). 7 individuals were assigned female at birth, and 1 individual was assigned male at birth. Non-Hispanic White (65,6%), Hispanic or Latina (3,2%), African American (4,5%, Asian American (2,6%), and multiracial (24%)	14–17	Experiences of social support and how it relates to well-being and resilience among transgender youth.	Support is acceptance with actions Taking supportive action steps Navigating the changing nature of support	Thematic analysis (Saldaña, 2016).
17.	Wilson et al. (2005)	The interaction between young people with atypical gender identity organization and their peers	UK	All participants were attending a Gender Identity Development Unit (GIDU), a specialist nationwide service for children and adolescents with gender identity problems. The interviews occurred the next time the young person was due to attend the GIDU, except for three participants who were interviewed at their homes, as they were not due to attend the GIDU in the immediate future.	*N* = 8 children with atypical gender identity organization; male to female (6) (MtF), female to male (2) (FtM]). Race/ethnicity was not specified.	14–17	The participants’ interaction with their peers in secondary school.	Bullying and homophobic abuse Peer support Disclosure	Thematic analysis (Smith, 1995).

## Results

A total of 2153 studies were retrieved after duplicates had been removed. See Figure 1 for information.

Eighteen articles met all SPIDER inclusion criteria and were assessed for quality.

### Study characteristics

The synthesis comprised 17 articles, encompassing 243 TGD youth aged 7–18. Among these articles, seven were conducted in the US, four in Canada, one from a collaborative effort between the US and Canada, three in the UK, and two in the Netherlands. Notably, four articles shared overlapping participant samples: specifically, two from Budge et al., 2018 and 2021, and two from Pullen Sansfaçon et al., 2019 and 2020). For Study features, see Table 2.

### Synthesis

See Table 3.

**Table 3.** Themes and sub-themes.

Theme	Sub-theme	Articles
Navigating gender	*Meaning-making*	Clark et al., 2020; Durwood et al., 2022; Katz-Wise et al., 2020; McGowan et al., 2022; McKenna et al., 2022; Saltis et al., 2023; Selkie et al., 2020: Steensma et al, 2011; Pullen Sansfaçon et al., 2020; Vrouenraets et al., 2016; Wilson et al., 2005
	*Considering visibility*	Budge et al., 2021; Clark et al., 2020; Durwood et al., 2022; Katz-Wise et al., 2020; Leonard, 2022; McGowan et al., 2022; McKenna et al. 2022; Pullen Sansfaçon et al., 2019; Pullen Sansfaçon et al., 2020; Selkie et al., 2020; Weinhardt et al., 2019; Wilson et al., 2005
Navigating relations	*Longing for belonging*	Budge et al., 2021; Katz-Wise et al., 2020; Kelley et al., 2022; Leonard, 2022; McGowan et al., 2022; McKenna et al, 2022; Saltis et al., 2023; Selkie et al., 2020
	*Supportive actions*	Budge et al., 2018; Clark et al., 2020; Katz-Wise et al., 2020; Kelley et al., 2022; Leonard, 2022, McGowan et al., 2022; Pullen Sansfaçon et al., 2019; Selkie et al., 2020; Weinhardt et al., 2019; Wilson et al., 2005
	*Lack of safety*	Budge et al., 2021; Kelley et al., 2022; McGowan et al., 2022; McKenna et al., 2022; Selkie et al., 2020; Vrouenraets et al., 2016; Weinhardt et al., 2019; Wilson et al., 2005
	*Coping inside out*	Budge et al., 2018; Clark et al., 2020; Katz-Wise et al., 2020; Kelley, et al., 2022; Leonard, 2022; McGowan, et al., 2022; Pullen Sansfaçon et al., 2019; Saltis et al., 2023; Weinhardt et al., 2019; Wilson et al., 2005
Navigating society	*Inclusion and exclusion*	Kelley et al., 2022; Leonard, 2022; McGowan et al., 2022; Selkie et al., 2020; Vrouenraets et al., 2016
	*Beyond control*	Budge et al., 2021; Clark et al., 2020; Katz-Wise et al., 2020; Pullen Sansfaçon et al., 2019; Steensma et al., 2011; Vrouenraets et al., 2016

#### Navigating gender identity

The first theme, “Navigating gender identity,” involved two sub-themes. The first, *Meaning-making,* explored TGD youth’s various gender identity journeys, initially trying to make sense of their feelings of dissonance by experimenting and seeking information to eventually achieve self-understanding in a larger context. In the second sub-theme, *Considering visibility*, the tension between stressful feelings of gender incongruence and fear of other people’s reactions, if they come out, was described as a central dilemma.

##### Meaning-making

TGD youth underwent diverse and individual journeys in developing their gender identity (Clark et al., 2020; Durwood et al., 2022; Katz-Wise et al., 2020; McGowan et al., 2022; McKenna et al., 2022; Pullen Sansfaçon et al., 2020; Steensma et al., 2011; Wilson et al., 2005). Their gender paths varied in timing, duration, and trajectory. A common thread among them was the confusing dissonance between their internal sense of self and the external cues from their bodies or social norms related to gender. Some children showed and expressed this early on in their lives (Durwood et al., 2022; Pullen Sansfaçon et al., 2020; Steensma et al., 2011). Even though the feeling of dissonance had been present, others became aware of it later. For certain individuals, gender was not a significant consideration during childhood until triggers like puberty, sexual attraction, or increasing societal expectations (Clark et al., 2020; Katz-Wise et al., 2020; McGowan et al., 2022; McKenna et al., 2022; Pullen Sansfaçon et al., 2020; Steensma et al., 2011; Wilson et al., 2005).

Initially, many TGD youth experienced a lack of comprehension about their feelings and reactions and, therefore, were not able to talk about them:

I think that I actually I still couldn’t fully understand it myself, I couldn’t really put a name to it or anything like that and it was and it would have been difficult to talk to people about it.” 16-year-old trans girl. (Wilson et al., 2005, p. 311)

Making sense of these inner experiences motivated youth to seek information (Clark et al., 2020; Katz-Wise et al., 2020; Pullen Sansfaçon et al., 2020). A phase of self-exploration, spanning from weeks to years, during which they searched, experimented, and pondered their gender identity before coming out to others was commonly described (Clark et al., 2020; Katz-Wise et al., 2020; McKenna et al., 2022; Pullen Sansfaçon et al., 2020). Many kept their information-seeking activity hidden, wanting to be certain. Fear of negative parental reactions to their exploration was also mentioned as a cause of secrecy. Increasing visibility in media (television programs, films, magazines, and newspapers), as well as medical information and other trans people’s stories online, lead youth to increased self-understanding and seeing their own gender identities more clearly (Clark et al. 2020; Katz-Wise et al., 2020; Pullen Sansfaçon et al., 2020; Selkie et al., 2020).

I feel like it’s hard to meet trans people and learn things about trans people even if you are trans, just in day-to-day interaction because one, there aren’t that many and two, a lot of trans people don’t really want you to know that they’re trans, and that makes it hard to start a conversation, and I can’t blame them because I’m in that boat. But social media because there’s more anonymity there really, more people are open to talking about their experiences as trans people and helps you understand it more. (Selkie et al., 2020, p. 277)

Some participants played video games and used avatars early on in their gender journeys to experiment with gender identities virtually without thinking about it too seriously, but it also helped some to understand and feel more comfortable with themselves in their gender identity (McKenna et al., 2022). Engaging with broader trans communities online could be an introduction to diverse gender identities and the realization they were not alone in their experiences. Learning more about trans-related and intersecting factors within a broader context, including oppressive forces and concurrent stressors, also played an important role in their process of meaning-making and self-understanding (Clark et al., 2020; Katz-Wise et al., 2020; McGowan et al., 2022; Saltis et al., 2023; Selkie et al., 2020: Vrouenraets et al., 2016).

For TGD youth, the journey of gender identity did not necessarily reach a definitive endpoint following their initial social transition (Durwood et al., 2022). It could involve multiple social transitions even before puberty if their internal sense of gender evolved over time or they discovered a more suitable identity, like a nonbinary, instead of the binary (boy/girl) (Durwood et al., 2022). These experiences underscore the fluid and intricate nature of gender identity (Durwood et al., 2022; McGowan et al., 2022; McKenna et al., 2022; Pullen Sansfaçon et al., 2020). Pursuing self-understanding and self-acceptance was an ongoing process as individuals navigated diverse paths to authentically express themselves.

##### Considering visibility

While the initial awareness of TGD youth’s gender identity was felt from within, relational factors were highly intertwined with the youth’s gender identity processes, affecting their considerations to come out to others (Budge et al., 2021; Durwood et al., 2022; Katz-Wise et al., 2020; Leonard, 2022; Pullen Sansfaçon et al., 2019; Weinhardt et al., 2019). Parental cues about the possibility of being accepted or not influenced the timing of their coming out, with fear of rejection potentially delaying the process. A 14-year-old girl said:

Because I couldn’t tell my mom when I was younger, I felt like I was in chains, where inside my mind, I couldn’t tell her. My fear was she was not going to be accepting. I was afraid that she was not going to let me be who I was. I was afraid that I was always going to remain a boy and being trapped into this person that I am not and being forced to play a part I’m not supposed to play. (Katz-Wise et al., 2020, p. 9)

Feelings of shame, anxiety, worry, and nervousness commonly accompanied the decision to come out. Dependence on caregivers emotionally, practically, legally, and financially led youth to adjust and compromise at the expense of their own needs (Budge et al., 2021; Katz-Wise et al., 2020; Leonard, 2022; Pullen Sansfaçon et al., 2019; Weinhardt et al., 2019).

On the other hand, other children showed early signs that their gender identity did not align with their assigned sex at birth through non-normative behavior and preferences and often expressed it verbally as well. These children typically received support from their parents, allowing for an early social transition by changing their name and pronouns (Budge et al., 2021; Durwood et al., 2022; Katz-Wise et al., 2020; Pullen Sansfaçon et al., 2020), as well as for possible social retransitions (Durwood et al., 2022) as this child entering the study as a trans girl but currently identifying as a boy—his sex assigned at birth:

They [youth’s parents]…asked me, I can go back to being a boy whenever, and I still said, “no, I still feel like a girl.” But then when I got older to now, I felt more—I noticed and felt more like a boy. 9-year-old boy (Durwood et al., 2022, p. 7)

Some TGD youth came out online before disclosing to family, friends, and at school (McKenna et al., 2022; Selkie et al., 2020). They described the paradox of being able to be more personal in the more impersonal environment online, having distance. Coming out online was often a first step, helping them get the confidence to take further steps in real life (McKenna et al., 2022; Selkie et al., 2020). For some, building avatars in online video games served as a low-pressure method of expressing or changing their gender identity without being questioned, providing a secure space for exploration (McKenna et al., 2022).

Navigating school environments was important in TGD youth’s coming out process (McGowan et al., 2022; Wilson et al., 2005). Careful consideration was given to the attitudes toward gender diversity at their school, influencing the timing and manner of their disclosure to maximize acceptance (McGowan et al., 2022). In some cases, youth perceived disclosing their gender identity as an immense risk that could potentially lead to losing their only friends, influenced by peer pressure and societal gender norms (Wilson et al., 2005).

Puberty was a significant turning point for many, triggering distress from unwanted bodily changes and intensifying gendered expectations (Clark et al., 2020; McGowan et al., 2022; Pullen Sansfaçon et al., 2019). Fear of other people’s reactions could lead TGD youth to not disclose until their distress from gender dysphoria and hiding their gender identity got so intense that it overruled the fear of being rejected (Clark et al., 2020; McGowan et al., 2022; Pullen Sansfaçon et al., 2019). This was expressed like this by one participant:

I started thinking about maybe I should come out because pronouns and that started to get on top of me and being called like a boy and stuff like that was really affecting me. (McGowan et al., 2022, pp. 32–33)

#### Navigating relations

In the second theme, “Navigating relations,” four sub-themes were identified. The first, *Longing for belonging,* highlighted the cruical role of recognition of gender identities for TGD youth’s sense of belonging. The second sub-theme, *Supportive actions*, emphasized the positive effect of others using chosen names and pronouns on TGD youth’s well-being, and the support of trans communities prioviding a respite from navigating gender-related social stress. The third, *Lack of safety*, explored the negative effects of constant vigilance for safety issues on mental health, well-being and school-related aspects. The fourth sub-theme, *Coping inside out*, described a bidirectional coping process where TGD youth’s inner sense of gender interacted with relational recognition and societal norms, and was modified by maturation, experience, creativity, and knowledge.

##### Longing for belonging

Accepting and validating relationships affirming the youth’s gender expressions and identities fostered positive emotions and a sense of belonging. When met with positive responses to coming out, such as when people used correct names and pronouns affirming their gender, youth experienced relief, happiness, and a sense of closeness to others (Budge et al., 2021; Katz-Wise et al., 2020; Kelley et al., 2022; Leonard, 2022; McGowan et al., 2022; McKenna et al., 2022). On the contrary, TGD youth felt sad when describing rejection from family and friends, for example, when being misgendered, as well as anger when thinking about invalidation/questioning of their gender identities (Budge et al., 2021). To be recognized by others was described as a deeply existential experience connected to being more valid as human beings (Weinhardt et al., 2019). Some youths also underlined the importance of being seen as a whole person, as a human, and not just as their gender identity (Saltis et al., 2023), ass described by this young person:

I like the gender-pointed questions. But um, I also like answering questions, as kind of me as a whole person. Because even though this thing is about my gender identity, I also want to just like, talk about my experience as a person, like who has this or who’s experienced this blah, blah, blah, not as a result of this. 15-year-old, genderfluid (Saltis et al., 2023, p. 15)

Many of the binary youths in the studies were not identified as “trans male” or “trans female” but simply as male or female (Budge et al., 2021; McGowan et al., 2022; Pullen Sansfaçon et al., 2019; Selkie et al., 2020). For them, “passing” as their identified gender was of major concern, strongly affecting them both ways. Positive feedback on their appearance and successful passing was considered validating (Budge et al., 2021; McGowan et al., 2022; Pullen Sansfaçon et al., 2019; Selkie et al., 2020). This could be expressed as:

Before I came out we were flying down to [location] and the airplane guy who was checking in our bags said “ma’am, come along with your son” and that was before I came out and I had just had my hair cut and my mom said that that whole week we were down there people were calling me boy and she said that I was smiling and way happier than I [had been]. 11-year-old, trans boy (Budge et al., 2021, p 156)

TGD youth appreciated engaging in ordinary conversations and creative activities with peers, especially with other trans kids where gender did not have to be an issue at all (Budge et al., 2018; Katz-Wise et al., 2020; McGowan et al., 2022; Saltis et al., 2023). For youth who had not come out “offline,” contact with online peers could help decrease feelings of isolation by finding belonging in an online community (Selkie et al., 2020). The importance of finding their own relaxing, nurturing space, whether in trans community groups, at Student Associations with Gay-Straight Alliances (GSAs), in creative contexts, or maybe online, was underlined by youth, as well as “being their own best friend” (Budge et al., 2018; Katz-Wise et al., 2020; Kelley et al., 2022; Leonard, 2022; McKenna et al., 2022; Saltis et al., 2023).

##### Supportive actions

Some youth defined support as a combination of acceptance and supportive actions demonstrating love and understanding for who they truly are, helping them affirm their gender identity, and facilitating transition (Weinhardt et al., 2019). While acceptance was the needed base, it was not sufficient. Supportive action steps could include parents informing school staff, helping them to buy new clothes after coming out, or standing up for them if they were mistreated. It could also be to seek relevant information and to help access medical and social services. In this way, the youth’s support needs shifted during their developmental and transitional journeys related to their gender identity (Katz-Wise et al., 2020; Weinhardt et al., 2019).

Some youth described their parental support as multifaceted, not always reliable, but marked by shifts or gradual development (Clark et al., 2020). For example, youth mentioned not getting support from parents regarding gender-affirming care but were still maintaining a supportive relationship with parents in other areas. Others regarded their parent’s unsupportiveness as possibly changing over time when they had processed and adjusted to their child’s gender identity (Clark et al., 2020; Weinhardt et al., 2019). One youth described this waiting period until getting support from their parents:

It takes time. I knew I would be safe, but I don’t feel supported. It just took time. I mean it’s rough getting misgendered by your parents for 2 years. I knew that I wouldn’t have an issue, even though it was scary. But it just took time. (Weinhardt et al., 2019, p. 321)

Beyond a supportive network of family and friends, support from teachers and peers at school carried paramount importance for youth’s well-being as school was another place of daily life that youth could not choose to get away from (Weinhardt et al., 2019). Peers and teachers using the right name and pronouns, as well as being humble about their own accidental pronoun mistakes by apologizing in a way, not increasing attention to the youth, were seen as supportive (Katz-Wise et al., 2020; Kelley et al., 2022; Leonard, 2022; McGowan et al., 2022). Adults’ attitudes and responses seemed to have a stronger impact on the youths’ well-being than the support from peers (Kelley et al., 2022), and individual teacher support was highlighted by youth as particularly important. When teachers educated themselves on gender issues or protected youth from being invalidated or bullied, resonated deeply with the youth (Leonard, 2022; McGowan et al., 2022). As one youth shared:

…she did say to teachers, ‘Listen’ (laughs), y’know, ‘that is not, a girl. This- that young man is a boy’. (Leonard, 2022, p. 50)

Social media was of great support for youth isolated from understanding peers or other TGD youth in the area (Selkie et al., 2020). Youth could find support in managing gender dysphoria, seeking information, and finding encouragement from those who had already navigated medicinal transitions (Selkie et al., 2020).

Most TGD youth had positive experiences from their contact with healthcare providers (Clark et al., 2020; Pullen Sansfaçon et al., 2019). Communication between clinic staff and parents to inform about medical issues and to ensure optimal support was valued by youth (Clark et al., 2020; Pullen Sansfaçon et al., 2019). Their gender clinics emerged as safe places for openly discussing the difficulties they experienced and obtaining guidance on hormone therapy, even if the need for information and support decreased as medical interventions started (Clark et al., 2020; Pullen Sansfaçon et al., 2019). Instead, some youth shared their experiences with others as a supportive way of giving back to their communities (Clark et al., 2020).

##### Lack of safety

TGD youth who felt safe could more easily disclose their gender identity, but many still feared for their safety in public spaces after coming out (Budge et al., 2021; Kelley et al., 2022). Physical school environments, especially their gendered facilities like locker rooms and bathrooms, were considered unsafe (Budge et al., 2021; Kelley et al., 2022). As TGD youth students rarely were consulted on issues concerning gendered locations, school solutions sometimes resulted in uncomfortable situations, such as a bathroom far off, or solutions leading to a higher level of discrimination (Kelley et al., 2022; McGowan et al., 2022).

Confidentiality was essential in fostering a sense of psychological safety for TGD youth (Kelley et al., 2022). If personal information in documents is not kept safe, it could force unwanted, accidental disclosure, undermining youth’s feeling of security. Respectively, maintained confidentiality was a protective factor in that safety enhanced youths’ well-being (Kelley et al., 2022; McGowan et al., 2022). One participant shared:

It’s really hard to show my papers to others, like people I just met. They have to check my deadname. For me, my deadname is something really private […]. 17-year-old, nonbinary (Kelley et al., 2022, p. 6)

In social contexts, numerous situations and behaviors had a negative impact on TGD youth’s well-being (Kelley et al., 2022; McGowan et al., 2022; Wilson et al., 2005). Experiences ranged from subtle invalidation to overt aggression. Rejection, particularly at school, was experienced by youth. From transphobic remarks, invasive and too intimate questions to refusal to use youth’s preferred names/pronouns (deadnaming and misgendering) and friends deliberately disclosing TGD youth to others without consent, were all common (Kelley et al., 2022; McGowan et al., 2022; Wilson et al., 2005). Verbal and physical bullying were also reported, sometimes subsiding after coming out (McGowan et al., 2022). Still, as stated in the study by McGowan et al. (2022) focusing on youth’s experiences in secondary school, no participant reported a complete absence of hostility.

Consequences of invalidation, exclusion, rejection, and bullying at school described by youth had a significant negative impact on their mental health and well-being, school attendance, and academic performance (Kelley et al., 2022; McGowan et al., 2022). This was expressed in this excerpt:

(Interviewer) “Is that anxiety having quite a big impact on your day-to-day school life?”; (Participant)” Mm hmm”; (Interviewer) “What kind of impact?”; (Participant) “I don’t go”. (McGowan et al., 2022, p 18)

Despite its utility for information and support, social media has a darker side. TGD youth described having had personal experiences of harassment and openly expressed transphobia online. Witnessing hateful comments directed at others could also be distressing (Selkie et al., 2020).

##### Coping inside and out

Navigating gender identity could be emotionally taxing for TGD youth. Many experienced difficult emotions, ranging from confusion and frustration to overwhelming stress and despair (Budge et al., 2018). Initially, many youths described silently conforming to societal expectations and trying to fit in by concealing their true selves (Budge et al., 2018; McGowan et al., 2022; Wilson et al., 2005). Efforts to ignore and avoid gender-related emotions before coming out sometimes culminated in crying to get some emotional release. For some, managing intense emotions could lead to more extreme strategies such as suicidal ideation, self-harm, or violent outbursts (Budge et al., 2018; Pullen Sansfaçon et al., 2019; Wilson et al., 2005). Over time, TGD youth developed emotionally mature ways to address their gender identity needs. Strategies included self-soothing techniques, creative self-expression, and self-advocacy skills, drawing support from family, peers, mentors, or healthcare providers when needed (Budge et al., 2018; Weinhardt et al., 2019). Engaging in self-care, creative, and joyful activities (Budge et al., 2018; Saltis et al., 2023 was important to cope with mental health issues and stressors related to gender and intersecting identities (Saltis et al., 2023) as well as trying to surround themselves supportive people (Kelley et al., 2022). The desire to contribute to positive change for other trans individuals grew as youth could assert themselves better. The goal broadened beyond their own well-being to active engagement through acts of advocacy, signifying a shift toward empowerment (Budge et al., 2018; Clark et al., 2020; Katz-Wise et al., 2020; Leonard, 2022; Saltis et al., 2023).

Coping with daily invalidating relationships posed dilemmas since it was impossible for TGD youth to always have supportive persons around, such as at school. They chose strategies for managing invalidating relations depending on the context and circumstances. Responses ranged from ignoring or actively distancing oneself from disrespectful peers to confronting them head-on (Budge et al., 2021; Kelley et al., 2022). Some used humor to counteract negativity or when facing barriers (Leonard, 2022). Online negative comments were reframed as opportunities for education (Selkie et al., 2020). At school, similar strategies were described, such as educating, raising awareness, and sensitizing others about gender diversity (Budge et al., 2021; Kelley et al., 2022). For some, this was also experienced as empowering:

I was invited to speak in front of some [SCHOOL] teachers- there’s 40 of them, I think- to discuss transgender, and how they can make the schools more accepting, and just answer any questions that teachers had I’ve done it for other kids who are transgender, who are younger, and had questions, or parents and other people… I think it was an honor. I like to help, and so it was cool that I could help in that way, especially since I went to [SCHOOL]. (Katz-Wise et al., 2020, p. 12)

Notably, a single strategy could yield varying outcomes based on context, timing, and purpose, underlining the importance of acknowledging the nuances of coping (Budge et al., 2021; Kelley et al., 2022). For instance, concealing one’s gender identity could be protective in hostile environments as well as stressful when denying authenticity before coming out (Budge et al., 2018; Clark et al., 2020; Katz-Wise et al., 2020; Kelley et al., 2022; McGowan et al., 2022; Weinhardt et al., 2019; Wilson et al., 2005). Another example was that advocacy and education efforts could foster pride and resilience, yet also exhaust youth if their voice remained unheard (Saltis et al., 2023).

#### Navigating society

The third theme, “Navigating society” consists of two sub-themes. The first, *Inclusion and exclusion,* enlightened how TGD youth’s sense of inclusion was deeply impacted by cis-normative gender norms, insufficient knowledge about gender diversity in schools, and stereotypical media portrayals of TGD individuals. Nonbinary identities were often overlooked. In the second sub-theme, *Beyond control*, TGDyouth described facing challenges beyond tpersonal and familial control, particurarly within healthcare systems at both organizational and national levels.

##### Inclusion and exclusion

Hetero- and cisnormativity passively reinforce gender norms via, for example, a gendered school system, facilities, and language (Kelley et al., 2022; McGowan et al., 2022; Vrouenraets et al., 2016). Since many TGD youths saw gender as a continuum rather than a binary system, particularly the nonbinary youth could feel disregarded and excluded. While it was seen as positive that laws have changed in a way that makes it possible to change the gender on official documents, this possibility could still be experienced as imposing a binary concept of gender on transgender individuals (Kelley et al., 2022; McGowan et al., 2022; Vrouenraets et al., 2016).

TGD youth perceived a generalized lack of knowledge as a significant barrier to being affirmed in their identity, resulting in inappropriate cis-normative comments from both peers and teachers. Even if remarks were not always intentional, they were rooted in stereotypical assumptions about gender and could be experienced as difficult to live with (McGowan et al., 2022; Kelley et al., 2022). Voicing the impact of these assumptions, a participant shared:

[…] people will judge that something is feminine then they’ll associate feminine with girl, that’s more difficult […] these things really hurt me, but others, you know, they can’t understand that, like, how we really feel. 17-year-old nonbinary person (Kelley et al., 2022, p. 5).

Recognizing the profound influence of school climate and culture on TGD youth’s well-being, they advocated for increased gender and sexual diversity education. They also regarded initiatives for positive school climate change on a whole-school level as essential to enhance understanding and acceptance of LGBTQ + individuals (Kelley et al., 2022; Leonard, 2022; McGowan et al., 2022). Youth expressed that this would enable them to spend less time feeling different and that it would be possible to explain their emotions if there was a shared understanding through language and knowledge of gender and sexual diversity by both pupils and staff (McGowan et al., 2022). TGD youth were mostly positive about the increased media coverage of transgender people but raised an aspect of exclusion in the often-stereotypical picture shown in media. For example:

Most of the trans men I have seen in the media…when they were younger, they always were stereotypical tomboys. I was personally quite stereotypically feminine; I liked drawing dresses with my mom, and still do. That made me feel alienated by the media. 18-years-old trans boy. (Vrouenraets et al., 2016, p. 1700)

Even inside transgender communities online, there could be posts about the “right” (binary) way to be transgender, which could feel invalidating for nonbinary youth (Selkie et al., 2020).

##### Beyond control

Numerous barriers lay beyond the control of TGD individuals and their families as they sought help and support. Prolonged waiting times from referral to the first appointment at a gender clinic, often spanning over a year, emerged as a major barrier (Clark et al., 2020; Pullen Sansfaçon et al., 2019). It also marked the culmination of an extended journey, starting with the youth’s internal quest for their gender identity, sometimes taking many years to come out, followed by a process within the family, where parents gradually recognized their children as they experienced themselves. Considering this, extended waiting times after finally seeking care could be extremely challenging (Clark et al., 2020; Pullen Sansfaçon et al., 2019). Youth reported having mixed feelings of frustration about waiting and hope about the further transition process (Budge et al., 2021).

Parents mostly initiated the decision to seek gender-affirming care, and some youths described a fear of burdening their parents with their medical needs (Clark et al., 2020; Pullen Sansfaçon et al., 2019). Consequently, youth with the lowest parental support experienced more system barriers (Clark et al., 2020). Unsupported youth described loneliness in navigating healthcare systems and longer delay times than supported youth before parents got involved. This paradox was frustrating, as information and support from professionals were often required before parents were ready to get on board (Clark et al., 2020).

Other barriers to care for TGD youth and their families were geographic factors that sometimes led to considerable distances to find qualified therapists, support groups, and medical care (Clark et al., 2020; Katz-Wise et al., 2020). Family financial constraints and lack of insurance coverage for gender affirmation in countries where this was required were yet others. The stress of coping with financial, bureaucratic, and legal landscapes was described as a strain.

The broader influence of the religious and political climate ultimately affected societal attitudes, school climate, laws regarding legal rights for transgender individuals, and recommendations for gender-affirming care (Clark et al., 2020; Katz-Wise et al., 2020 ). Support could be hard to find as expressed by this 15-year-old boy:

Living in [STATE] it is kind of hard to find different people who are like, there for support. I went to three or four different counselors before I found [THERAPIST] in [CITY] who is a ways away, but very good, and my endocrinologist is an hour away and all the surgeons that are good are three or four hours away. So, my support in that sense, is hindered because of my distance, in the middle of nowhere. (Katz-Wise et al., 2020, p. 25)

Youth sought gender-affirming medical care mainly to reduce their suffering from gender dysphoria. “Feeling down” or actual depressive feelings linked to their gender dysphoria were also described by youth recruited from gender clinics, and several mentioned having had suicidal thoughts, having self-harmed, or having engaged in self-induced vomiting and food restriction (Clark et al., 2020; Pullen Sansfaçon et al., 2019; Steensma et al., 2011). One participant described:

The dysphoria is getting so bad that I feel self-conscious all the time… I’m graduating in a year and a half, and I’ve realized I can’t be in this same position… I needed to sort of fix that problem in my head before it was too late to the point where I was back in that dark spot and I didn’t know how to get out. (Clark et al., 2020, p. 140)

Youth emphasized the importance of puberty blockers to prevent unwanted secondary characteristics and to allow time for decisions on gender-affirming hormonal treatment. To be able to pass and be read as their identified gender in the future was highly valued and hoped for (Pullen Sansfaçon et al., 2019). Even if suffering from gender dysphoria was hard to handle, TGD youth did not necessarily seek extensive interventions to change their (whole) body in the future. Most wanted hormonal treatment and anticipated positive outcomes. However, nonbinary youth were often unsure of how hormones would help them with the bodily changes they felt were needed to feel affirmed in their gender, compared to binary-identified trans youth:

[Blockers is] a maximum two years. And then I have to choose if I want to go on estrogen or testosterone. And that’s again that I feel I’m more middle. I’m not, I don’t want to choose one. I just want to stay at my body, which is not completely done. I’m stopped. 16-year-old trans male, nonbinary person (Pullen Sansfaçon et al., 2019, p. 378)

Expressing apprehension toward medical procedures, most youths were positive to research evaluating medical treatment, such as the impact of puberty suppression on bone mineral density (Pullen Sansfaçon et al., 2019; Vrouenraets et al., 2016). Balancing potential long-term effects of gender-affirming medical care with limited current data, youth weighed risks against the well-being they found in embracing their authentic selves, as this 14-year-old trans girl expressed it:

I would rather live 10 years shorter but live a very happy life being myself, than live 10 years longer but be unhappy my whole life. (Vrouenraets et al., 2016, p. 1700)

Moments of questioning their medical transition were part of the youth’s transition process, but none of the participants reported regrets (Clark et al., 2020; Pullen Sansfaçon et al., 2019).

## Discussion

The aim of this qualitative systematic review was to explore and synthesize TGD youth’s lived experiences and coping related to gender identity. Three overarching themes were identified through thematic analysis: (1) “Navigating gender” revealed TGD youth’s complex and unique journeys as they internally and externally navigated their gender identity. The results highlighted the fluid and dynamic nature of gender development among the younger generation. (2) “Navigating relations” underscored the significance of establishing a sense of belonging through the validation, recognition, and support of TGD youth’s identities within their social contexts, and highlighted concerns about safety in various settings. Many found solace and understanding within trans communities, providing support from the ongoing stressors tied to social cues regarding gender identity. (3) “Navigating society” shed light on the challenges posed by traditional gender norms, leading to feelings of exclusion. Gendered language, lack of knowledge about gender diversity, and stereotypical media portrayals contributed to TGD youth’s experiences of marginalization. Beyond personal control, societal barriers to accessing gender-affirming care and broader societal attitudes influenced by political and religious factors, added further frustration and distress in TGD youth.

The second research question was how TGD youth cope with their experiences. Coping was found to be context-dependent, dynamically influenced by factors, such as individual sense of gender, maturation, accumulated experiences, knowledge and creativity, recognition from relationships, and societal norms. The coping strategies were not fixed but evolved over time and were used actively and adaptively by TGD youth.

The majority of included studies (*n* = 14) were published between 2018 and 2023, indicating the rapid growth of qualitative research focusing on TGD youth 18 years or younger. In research with TGD youth, knowledge production and societal change regarding gender identity is moving fast, but descriptions from TGD youth interviewed themselves are still very limited. Particularly, only three of the study populations (Budge et al. 2018, 2021, and Pullen Sansfaçon et al. 2019, 2020, and Durwood et al. 2022) included (prepubertal) children under 13 years. This is a group that we will most probably hear more about in future research. They are the first generation TGD children now growing up in many Western countries who live socially transitioned from an early age with support from their parents.

In the first theme, “Navigating gender,” the youth described their ongoing gender identity development as a fluid process with non-linearity and variations (Durwood et al., 2022; McGowan et al., 2022; McKenna et al., 2022; Pullen Sansfaçon et al., 2020). This aligns with Jessen et al. (2021),,the previous review closest to ours regarding sample and research questions. Their results, in line with ours, emphasize the gradual negotiation of gender identity where feelings of dissonance and being different from cisgender youth were influenced by exploration, accumulated knowledge of gender diversity, social interactions, and increasing social demands. However, the role of the body and sexuality was more openly expressed by samples in Jessen et al. (2021) compared to the ones in our studies. This can possibly be explained by our younger samples and their stronger focus on gender dysphoria. Durwood et al. (2022) described some TGD youth who had experiences of several early social (re-) transitions in an ongoing process. This differs somewhat from earlier descriptions in the research literature. In the older study by Steensma et al. (2011), researchers defined "desisting" as a phenomenon where youth, who initially identified with one gender in childhood, later transitioned to a different gender identity. In this context, "desisting" meant that these individuals were no longer experiencing gender dysphoria and were returning to what was considered their original gender identity, as opposed to those who were "persisting" in their pursuit of a different gender identity. This interpretation suggests a binary perspective on gender identity and identity development. However, it is important to note that the study also acknowledged variations in how the two groups described their early gender identity experiences. Increased awareness of the spectrum of gender identities, including nonbinary identities, may influence how these findings are interpreted and reported today.

In the study by Durwood et al. (2022), the supportive but still emotionally quite neutral reactions reported by youth from family members, no matter the child’s gender identity, seemed crucial for gender identity exploration without experiencing rejection, distress, and regret (Durwood et al., 2022). The children seemed enabled to genuinely follow and freely express their unique gender identity paths in contrast to TGD youth, in several of the other studies, who described that they had to compromise and disregard their needs because of negative attitudes or considering the needs of others. Further, since as young children as prepubertal ones in Durwood et al. (2022) made retransitions, it seemed they did not feel they had to hide and seek information on their own when trying to make meaning of their experiences as many TGD youth in the other studies described. Drawing from the narratives in the other studies, where TGD youth described their sensitive perceptiveness to parents’ (and others’) emotional cues about their possible rejection, it is likely that prepubertal, young children also make an intuitive, emotional evaluation of their parents’ attitudes toward their diverse gender expressions, and that supportive and flexible responsiveness helps them through their different disclosures. Not making it a “big issue” seems like a helpful concept for TGD youth to enable an exploration of their gender identity without fear or shame, as well as getting support from significant persons. This approach is found to be most helpful in clinical work with Swedish TGD youth and their families (Kindstedt & Wurm, 2023).

In the second theme, “Navigating relations,” the relational dilemmas considering validation and safety were reported by youth as continuous navigation in daily life—in their families, at school, and sometimes when just being outdoors. This has further implications for the health of TGD minors as they cannot choose to stay away from many of these contexts. Other minority youth may also face additional challenges, but TGD youth often find themselves as the sole individuals within their family with their gender identity and one of few at school, making it more challenging to access support. In the older study included in our review by Wilson et al. (2005), all the participants had major doubts about coming out at school because of transphobic environments, and all but one reported being bullied. In the newer studies, TGD youth had somewhat more positive experiences. This might reflect some progress in schools toward a more inclusive environment, and/or it may be related to the fact that participants for Wilson et al. (2005) were recruited solely from gender clinics.

The possibility of rejection described by participants in our study was experienced and dealt with daily. Therefore, we consider this a lowkey stress and a type of constant emotional work, much like the kind of *emotional labor* (Hochschild, 1979) described in work environments. In line with this, youth also may have to hide or adjust their true feelings depending on societal gender norms and the experienced safety of the specific context. This daily emotional tension compounds the challenges they already face, rendering daily life more demanding than that of their peers adhering to societal norms. A unique challenge for TGD youth, even compared to other minority youth, lies in the disbelief, and invalidation of their identity and experiences, even by people close to them. While other minority youth can experience racism, and stigma due to functionality or social class, just as TDG youth experience transphobia, their existence as human beings is rarely questioned. Even if the Swedish sample in the study of Lundberg et al. (2023) consisted of transgender adults (17–63 years), we believe that the association made between minority stress, coping, and emotional labor could still be useful for a better understanding of the experiences of TGD youth, since minority stress theory and coping do not explain all nuances of exposure or TGD youth’s daily coping with social tension. Here it was highlighted how trans people had to carve out a livable space for themselves, something we can also see in the studies included. This possibly continuous drain of energy could be exhausting to deal with for TGD youth, in addition to the other developmental issues that they share with all young people, no matter their gender or sexual orientation. This also highlights the importance of finding relations and places for an emotional sense of belonging and where gender identity is not an issue. This was mentioned by TGD youth in some of our selected studies. Therefore, safe spaces can be understood not only in terms of social capital by increasing support and resistance to stigma, as mentioned in the review by Smith et al. (2021), but also possibly as health-enhancing by reducing stress hormones and making physiological as well as psychological recovery from daily emotional work possible. The included studies in the review of Sherman et al. (2020) on trans community connection found several links to positive outcomes in (mainly adult) transgender people, such as improved mental health, supported exploration of gender and sexuality identities, and informed gender transition. Still, the studies in the review focused mainly on the effects of *behavioral* participation, where authors noticed a “ceiling effect” when the amount of participation no longer affected the psychological distress. Only a few studies searched for aspects of connectedness in line with more emotional and existential experiences through an inner sense of belonging and calmness within oneself, described by Lundberg et al. (2023) as “minority peace.” The need for belonging is an intrinsic and profoundly experienced human need and motivator, prompting TGD youth to employ various strategies to increase their chances of acceptance. They actively sought information about gender, carefully timed their coming out, and adapted their approaches, sometimes even compromised their own needs. Understanding the dignity of this makes an existential dimension visible in youth’s stories. As such, we consider all our themes to include deeply existential dimensions of being a human, not sufficiently underlined in other reviews except for Jessen et al. (2021).

In the review of Johns et al. (2018), the authors found four protective skills/competencies for transgender youth on an individual level—personal mastery, ability to use the internet or social media for information or support, problem-solving skills, and self-advocacy. These results resemble the helpful coping described by TGD youth in our review. However, the bidirectional and developmental process of coping, where youth’s inner sense of gender, maturation, and accumulated experiences interacted with knowledge, relational recognition, and societal norms, becomes more evident through the synthesis of our studies. Highlighting the importance of context and nuances of coping is also crucial in understanding youth’s progression in caring for their health and needs. The review of Smith et al. (2021) on adaptive coping responses to minority stress did underline a progression from “social abuse to social action,” in line with the developmental aspect in our review, and the resilience built by actively engaging and taking charge of one’s health and needs. However, their review only looked at intentional coping in line with *proactive coping* (Biggs et al., 2017), where coping strategies are planned to prevent and handle possible stressors in upcoming situations, while we also described more immediate and reactive coping as being dominant in younger youth at the beginning of their gender journeys. The review of Smith et al. (2021) did not include minors, making our review an important contribution to describing youth’s development of coping skills as well as the competence of minors.

Since TGD youth experienced it as an especially important and immediate sign of respect and acceptance when people used their chosen names and pronouns, the importance of language for the acknowledgment and recognition of gender identity in society was highlighted. We read youth’s emotional struggles as existential strivings for being accepted as existing, legitimate human beings with the same need as others for representation through language, visibility, and safe societal places. This seems especially needed for TGD youth with nonbinary identities, as they they often lack acknowledgment, also evident in the lack of non-gendered activities and facilities, and in the oftentimes binary-gendered structure and interventions of gender-affirming care.

### Limitations and strengths

This review has certain limitations that need consideration. Firstly, several of the first authors of the articles included were coauthors in additional articles, and in some studies, the same sample was used, which limits the number of participants and potentially impacts the interpretation of results. It also points to the limited amount of research made in qualitative research, where minor TGD youth themselves have been heard through interviews.

Another limitation is the lack of diversity in participant demographics, specifically in terms of ethnicity/race, economic status, and parents’ educational background. In the few studies that included prepubertal children, the majority lived in white, well-educated, and economically middle-class families, limiting insights into the experiences of more diverse TGD child populations. All studies specifying race/ethnicity, identified participants as white, except for the study by Saltis et al. (2023), which had a somewhat more varied sample and an intersectional approach. Six of the studies did not specify the race/ethnicity of their sample at all. However, since all but one (in Canada) were conducted in Europe, we can only suspect that these samples were also predominantly white. A more diverse participant pool is important for improving relevant interventions for TGD youth from all societal levels and cultures, but also for raising knowledge in professionals and a broader audiance meeting TGD youth. Potential bias is also introduced by the requirement of parental consent for ethical approval when including children in research. This is obviously needed for the protection of minors but also affects the representation of TGD youth without family support in their gender journeys. Further bias stems from the geographical distribution of studies, where the majority was conducted in the US/Canada (12 articles, 184 TGD youths) and the rest in Europe (the Netherlands and the UK; 5 articles, 59 TGD youths). This limits the generalizability of findings due to variations in gender discrimination laws, organizational structures, access to gender-affirming care, and political influences on societal gender norms and attitudes.

Despite these limitations, the review has notable strengths. To our knowledge, it is the first to synthesize qualitative research specifically on the experiences and coping of TGD youth 18 years or younger. The acknowledgment of TGD minors’ voices, particularly those of prepubertal children, is a crucial contribution as their experiences are underrepresented in research. The youth in the included studies were recruited from diverse contexts, not solely from gender-affirming health care, providing a comprehensive perspective on TGD youth’s experiences since these may be affected by suffering from gender dysphoria and mental health.

Although there was a deviation from the initial time limit, a full, renewed, and updated, systematic search and selection process was conducted. The quality of primary studies was assessed according to nationally approved standards for qualitative research, and only good to moderate quality studies were included, enhancing the trustworthiness of the review. The collaborative approach, involving multiple authors working independently, regular reflexive discussions within the research group, and adherence to the ENTREQ statement for reporting guidelines, further strenghen the review. Additionally, the diverse representation of gender and sexual identities within the research group, coupled with clinical experience of working with youth and their families and conducting research with transgenderpeople adds credibility and depths to the study.

### Implications for future research

Future research should continue to prioritize including the voices of TGD minors, a group currently underrepresented in research. Addressing the challengeof reaching TGD youth without family support in their gender journeys may necessitateexploring alternative consent processes, ensuring ethical approval while safeguarding participant anonymity and confidentiality. Additionally, efforts should focus on exploring the experiences of TGD youth in diverse contexts using an intersectional approach. Conducting longitudinal studies to track TGD youth’s experiences over time can significantly contribute to our understanding of the fluid nature of gender identity. These studies can inform the development of relevant and effective interventions and support systems to promote the health and well-being of TGD youth, including youth with nonbinary gender identities.

### Conclusions

In conclusion, this systematic review of qualitative research literature provides a nuanced exploration of the experiences of TGD minors related to gender identity and coping. It expands the knowledge of youths’ gender identity processes as various, non-linear, and sometimes fluid with several social (re-)transitions, without valuing one as righter. TGD youth consider others’ use of their chosen names/pronouns as vital signs of respect and acceptance, and their struggles reflect existential yearnings for belonging, recognition, representation, visibility, and safe spaces. Nonbinary youth, in particular, face inadequate acknowledgment through gendered language, facilities, and gender-affirming care structures. Coping is described in its complexity and nuances as developmental aspects and social contexts influence strategies. The subtle daily and possibly exhausting emotional work of navigating microaggressions, and the psychological and existential dimensions of belonging for experiencing soothing "minority peace", are needed additions to minority stress theory and coping. Thus, this review can inform the practice of professionals working with TGD minors and give important information to families and other social arenas that include TGD minors.

### Implications

- Fast-Evolving Knowledge: The rapid growth of research focusing on TGD youth indicates the need for healthcare professionals to stay updated with the latest knowledge and best practices. Continued education and training are essential to provide effective care for this population.
- Early Social Transition: The emergence of socially transitioned prepubertal children highlights the importance of early support from parents. Clinicians should provide resources and guidance to parents to help them navigate their child’s gender identity development. However, it is essential for the society as a whole to recognize the significance of support and actively contribute to creating an atmosphere that embraces also TGD children.
- Recognition: Using chosen names and pronouns is a vital sign of respect for TGD youth and is crucialin fostering a sense of legitimacy and acceptance.
- Safety Concerns: TGD youth often face safety concerns in various contexts, such as school or public spaces. It is essential to create safe environments that protect TGD youth from physical and psychological harm. Anti-bullying programs and policies, increased knowledge of gender variance, and gender-neutral activities and facilities that promote inclusivity are crucial.

## Supplementary Material

Supplemental Material

Supplemental Material
