# Supplementary material for: A systematic review and qualitative research synthesis of the lived experiences and coping of transgender and gender diverse youth 18 years or younger
Source: Int J Transgend Health. 2024 Jan 12;25(3):352–88. doi: 10.1080/26895269.2023.2295379 (PMC11268253; doi:10.1080/26895269.2023.2295379)
Supplement: Supplemental Material [file WIJT_A_2295379_SM1328.docx]

# Appendix B

Unofficial translation of questions for assessment of qualitative studies from

*Bedömning av studier med kvalitativ metodik* ([*Assessment of qualitative studies*];

Statens beredning för medicinsk och social utvärdering [Swedish Agency for Health

Technology Assessment and Assessment of Social Services], 2020)

**1. Correspondence between philosophical approach/theory and sample**

**and method in the study**

• From which theory or philosophical approach did the authors

proceed?

• Does the research aim and question correspond to the theoretical/philosophical stance?

☐ Yes ☐ No ☐ Unclear

Comments:

**2. Participants**

• How was sampling done?

*Supporting questions in the assessment:*

• Is the sample suitable to answer the research question?

☐ Yes ☐ No ☐ Unclear

• Is the recruitment method suitably chosen and executed?

☐ Yes ☐ No ☐ Unclear

• Are there serious concerns that might influence the trustworthiness

of the study?

☐ Yes ☐ No ☐ Unclear

Comments:

**3. Data collection**

• Which methods were used in data collection?

• Are there serious concerns with the data collection that might

influence the trustworthiness of the study?

☐ Yes ☐ No ☐ Unclear

2

Comments:

**4. Analysis**

• Which methods were used for the analysis?

*Supporting questions in the assessment:*

• Is the chosen method for analysis suitable and suitably executed?

☐ Yes ☐ No ☐ Unclear

• Were the researchers reflexive in their interpretation of the data?

☐ Yes ☐ No ☐ Unclear

• Were the interpretations validated?

☐ Yes ☐ No ☐ Unclear

• Are there serious concerns with the analysis that might influence

the trustworthiness of the study?

☐ Yes ☐ No ☐ Unclear

Comments:

**5. Researcher(s)**

• Which background and competence did the researchers have?

*Supporting questions in the assessment:*

• Do the researchers have any kind of relationship with the

participants that might influence data collection?

☐ Yes ☐ No ☐ Unclear

• Have the researchers acted upon their fore-understanding in an

acceptable manner?

☐ Yes ☐ No ☐ Unclear

• Were the researchers independent of financial or other

considerations that might influence the analysis?

☐ Yes ☐ No ☐ Unclear

• Are there serious concerns that might influence the trustworthiness

of the study?

☐ Yes ☐ No ☐ Unclear

Comments:

**6. Relevance**

☐ The study is relevant

☐ The study is partially relevant

☐ The study is indirectly relevant

☐ The relevance cannot be assessed

Comments:

**7. Coherence**

*Supporting questions:*

• Was the main part of the data used in the analysis?

☐ Yes ☐ No ☐ Unclear

• Were contradictory data treated in a suitable manner?

☐ Yes ☐ No ☐ Unclear

• Did the collected data support the result?

☐ Yes ☐ No ☐ Unclear

• In summary, are there serious weaknesses that might lead to

insufficient coherence in the scientific basis of the study?

☐ Yes ☐ No ☐ Unclear

Comments:

**8. Sufficient data**

*Supporting questions:*

• Was the number of participants sufficiently great? (e.g., if

saturation had been achieved)

☐ Yes ☐ No ☐ Unclear

• Did the method of data collection allow for the possibility of rich data?

☐ Yes ☐ No ☐ Unclear

Comments:
