# Supplementary material for: A systematic review and qualitative research synthesis of the lived experiences and coping of transgender and gender diverse youth 18 years or younger
Source: Int J Transgend Health. 2024 Jan 12;25(3):352–88. doi: 10.1080/26895269.2023.2295379 (PMC11268253; doi:10.1080/26895269.2023.2295379)
Supplement: Supplemental Material [file WIJT_A_2295379_SM1327.docx]

# Appendix A

**URL to last completed search strategy [30 March 2023]**

***PsycINFO: 1434 results***

((ti,ab(("gender identities" OR "gender identity") OR transsexual* OR ("gender dysphoria") OR "gender incongruen*" OR transgender* OR trans OR ("gender variance" OR "gender variant") OR ("gender diverse" OR "gender diversity") OR "gender creativ*" OR "gender expansiv*" OR "gender nonconforming" OR ("social transition")) OR (MAINSUBJECT.EXACT("Gender Identity") OR MAINSUBJECT.EXACT("Gender Reassignment") OR MAINSUBJECT.EXACT("Gender Dysphoria") OR MAINSUBJECT.EXACT("Transgender") OR MAINSUBJECT.EXACT("Psychosexual Development") OR MAINSUBJECT.EXACT("Transsexualism") OR MAINSUBJECT.EXACT("Gender Nonconforming"))) AND (ti,ab(("lived experience" OR "lived experiences") OR ("life experience" OR "life experienced" OR "life experiences") OR attitude* OR view* OR perspective* OR experience* OR perception* OR coping) OR (MAINSUBJECT.EXACT("Explicit Attitudes") OR MAINSUBJECT.EXACT("Coping Behavior") OR MAINSUBJECT.EXACT("Social Perception") OR MAINSUBJECT.EXACT("First Experiences") OR MAINSUBJECT.EXACT("Self-Perception") OR MAINSUBJECT.EXACT("Life Experiences") OR MAINSUBJECT.EXACT("Strategies") OR MAINSUBJECT.EXACT("Coping Style") OR MAINSUBJECT.EXACT("Early Experience") OR MAINSUBJECT.EXACT("Role Perception") OR MAINSUBJECT.EXACT("Life Review") OR MAINSUBJECT.EXACT("Implicit Attitudes") OR MAINSUBJECT.EXACT("Sex Role Attitudes"))) AND (ti,ab(("qualitative studies" OR "qualitative study") OR "qualitative research" OR "qualitative design" OR "qualitative analysis" OR "qualitative data" OR ("qualitative method" OR "qualitative methodology") OR "qualitative descriptive stud*" OR "qualitative interview*" OR "content analysis" OR "thematic analysis" OR "grounded theory" OR ethnograph* OR ethnolog* OR phenomenograph* OR phenomenolog* OR hermeneutic* OR narrative* OR "nursing methodolog*" OR "sociological research" OR "discourse analysis") OR (MAINSUBJECT.EXACT("Content Analysis") OR MAINSUBJECT.EXACT("Grounded Theory") OR MAINSUBJECT.EXACT("Constructivism") OR MAINSUBJECT.EXACT("Mixed Methods Research") OR MAINSUBJECT.EXACT("Qualitative Measures") OR MAINSUBJECT.EXACT("Discourse Analysis") OR MAINSUBJECT.EXACT("Interpretative Phenomenological Analysis") OR MAINSUBJECT.EXACT("Focus Group") OR MAINSUBJECT.EXACT("Semi-Structured Interview") OR MAINSUBJECT.EXACT("Interviews") OR MAINSUBJECT.EXACT("Narrative Analysis") OR MAINSUBJECT.EXACT("Focus Group Interview") OR MAINSUBJECT.EXACT("Hermeneutics") OR MAINSUBJECT.EXACT("Phenomenology") OR MAINSUBJECT.EXACT("Qualitative Methods")) OR me.exact("Qualitative Study" OR "Interview" OR "Focus Group" OR "Clinical Case Study")) AND (su.exact("Childhood (birth-12 yrs)" OR "Adolescence (13-17 yrs)" OR "School Age (6-12 yrs)" OR "Preschool Age (2-5 yrs)") OR (MAINSUBJECT.EXACT("Early Childhood Development") OR MAINSUBJECT.EXACT("Early Adolescence") OR MAINSUBJECT.EXACT("Childhood Development") OR MAINSUBJECT.EXACT("Adolescent Development")) OR ti,ab(Adolescen* OR child* OR youth OR ("school age" OR "school aged" OR "school ages") OR ("preschool age" OR "preschool aged" OR "preschool ages") OR tween* OR teenager*)) AND (pd(20000101-20230331) AND PEER(yes))) AND PEER(yes)

***ASSIA: 243 results***

(ti,ab(("gender identities" OR "gender identity") OR transsexual* OR ("gender dysphoria") OR "gender incongruen*" OR transgender* OR trans OR ("gender variance" OR "gender variant") OR ("gender diverse" OR "gender diversity") OR "gender creativ*" OR "gender expansiv*" OR "gender nonconforming" OR ("social transition")) OR (MAINSUBJECT.EXACT("Gender identity") OR MAINSUBJECT.EXACT("Gender roles") OR MAINSUBJECT.EXACT("Gender identity disorder") OR MAINSUBJECT.EXACT("Crossgender behaviour") OR MAINSUBJECT.EXACT("Psychosexual development") OR MAINSUBJECT.EXACT("Transsexuality") OR MAINSUBJECT.EXACT("Gender dysphoria") OR MAINSUBJECT.EXACT("Gender norms"))) AND (ti,ab(("lived experience" OR "lived experiences") OR ("life experience" OR "life experienced" OR "life experiences") OR attitude* OR view* OR perspective* OR experience* OR perception* OR coping) OR (MAINSUBJECT.EXACT("Affective experiences") OR MAINSUBJECT.EXACT("Social experiences") OR MAINSUBJECT.EXACT("Cognitive coping") OR MAINSUBJECT.EXACT("Emotional experiences") OR MAINSUBJECT.EXACT("Subjective experiences") OR MAINSUBJECT.EXACT("Early life experiences") OR MAINSUBJECT.EXACT("Coping strategies") OR MAINSUBJECT.EXACT("Life experiences") OR MAINSUBJECT.EXACT("Childhood experiences") OR MAINSUBJECT.EXACT("Personal experiences") OR MAINSUBJECT.EXACT("Coping skills") OR MAINSUBJECT.EXACT("Experiences") OR MAINSUBJECT.EXACT("Emotional coping") OR MAINSUBJECT.EXACT("Beliefs") OR MAINSUBJECT.EXACT("Coping style") OR MAINSUBJECT.EXACT("Attitudes") OR MAINSUBJECT.EXACT("Daily experiences") OR MAINSUBJECT.EXACT("Coping") OR MAINSUBJECT.EXACT("Opinions"))) AND (ti,ab(("qualitative studies" OR "qualitative study") OR "qualitative research" OR "qualitative design" OR "qualitative analysis" OR "qualitative data" OR ("qualitative method" OR "qualitative methodology") OR "qualitative descriptive stud*" OR "qualitative interview*" OR "content analysis" OR "thematic analysis" OR "grounded theory" OR ethnograph* OR ethnolog* OR phenomenograph* OR phenomenolog* OR hermeneutic* OR narrative* OR "nursing methodolog*" OR "sociological research" OR "discourse analysis") OR (MAINSUBJECT.EXACT("Qualitative data") OR MAINSUBJECT.EXACT("Qualitative methods") OR MAINSUBJECT.EXACT("Content analysis") OR MAINSUBJECT.EXACT("Depth interviewing") OR MAINSUBJECT.EXACT("Qualitative analysis") OR MAINSUBJECT.EXACT("Interpretative phenomenological analysis") OR MAINSUBJECT.EXACT("Focus groups") OR MAINSUBJECT.EXACT("Discourse analysis") OR MAINSUBJECT.EXACT("Autobiographical interviewing") OR MAINSUBJECT.EXACT("Focus group interviews") OR MAINSUBJECT.EXACT("Interviews") OR MAINSUBJECT.EXACT("Hermeneutics") OR MAINSUBJECT.EXACT("Grounded theory") OR MAINSUBJECT.EXACT("Qualitative research") OR MAINSUBJECT.EXACT("Personal narratives") OR MAINSUBJECT.EXACT("Phenomenology") OR MAINSUBJECT.EXACT("Semistructured interviews"))) AND (ti,ab(adolescen* OR child* OR youth OR ("school age" OR "school aged" OR "school ages") OR ("preschool age" OR "preschool aged" OR "preschool ages") OR tween* OR teenager*) OR (MAINSUBJECT.EXACT("Young children") OR MAINSUBJECT.EXACT("Preschool children") OR MAINSUBJECT.EXACT("Adolescents") OR MAINSUBJECT.EXACT("Children") OR MAINSUBJECT.EXACT("Young people"))) AND (pd(20000101-20230331) AND PEER(yes))

***PubMed: 1,011 results***

(("Child"[MeSH Terms:noexp] OR "child, preschool"[MeSH Terms] OR "Adolescent"[MeSH Terms] OR ("adolescen*"[Title/Abstract] OR "child*"[Title/Abstract] OR "youth"[Title/Abstract] OR "school age*"[Title/Abstract] OR "preschool age*"[Title/Abstract] OR "tween*"[Title/Abstract] OR "teenager*"[Title/Abstract])) AND ("Gender Identity"[MeSH Terms:noexp] OR "Psychosexual Development"[MeSH Terms:noexp] OR "Gender Dysphoria"[MeSH Terms] OR "Transgender Persons"[MeSH Terms] OR "Transsexualism"[MeSH Terms] OR ("gender identit*"[Title/Abstract] OR "transsexual*"[Title/Abstract] OR "gender dysphori*"[Title/Abstract] OR "gender incongruen*"[Title/Abstract] OR "transgender*"[Title/Abstract] OR "trans"[Title/Abstract] OR "gender varian*"[Title/Abstract] OR "gender divers*"[Title/Abstract] OR "gender creativ*"[Title/Abstract] OR "gender expansiv*"[Title/Abstract] OR "gender nonconforming"[Title/Abstract] OR "social transition*"[Title/Abstract])) AND ("Life Change Events"[MeSH Terms] OR "Attitude"[MeSH Terms:noexp] OR "Emotions"[MeSH Terms] OR "adaptation, psychological"[MeSH Terms:noexp] OR ("lived experience*"[Title/Abstract] OR "life experience*"[Title/Abstract] OR "attitude*"[Title/Abstract] OR "view*"[Title/Abstract] OR "perspective*"[Title/Abstract] OR "experience*"[Title/Abstract] OR "perception*"[Title/Abstract] OR "coping"[Title/Abstract])) AND ("Qualitative Research"[MeSH Terms:noexp] OR "Hermeneutics"[MeSH Terms] OR "Focus Groups"[MeSH Terms] OR "Interviews as Topic"[MeSH Terms] OR "Autobiography"[Publication Type] OR "Personal Narrative"[Publication Type:noexp] OR "Interview"[Publication Type] OR "Grounded Theory"[MeSH Terms] OR ("qualitative stud*"[Title/Abstract] OR "Qualitative Research"[Title/Abstract] OR "qualitative design"[Title/Abstract] OR "qualitative analysis"[Title/Abstract] OR "qualitative data"[Title/Abstract] OR "qualitative method*"[Title/Abstract] OR "qualitative descriptive stud*"[Title/Abstract] OR "qualitative interview*"[Title/Abstract] OR "content analysis"[Title/Abstract] OR "thematic analysis"[Title/Abstract] OR "Grounded Theory"[Title/Abstract] OR "ethnograph*"[Title/Abstract] OR "ethnolog*"[Title/Abstract] OR "phenomenograph*"[Title/Abstract] OR "phenomenolog*"[Title/Abstract] OR "hermeneutic*"[Title/Abstract] OR "narrative*"[Title/Abstract] OR "nursing methodolog*"[Title/Abstract] OR "sociological research"[Title/Abstract] OR "discourse analysis"[Title/Abstract]))) AND (2000/1/1:2023/3/31[pdat])
